# Dental Stem Cell-Derived Secretome/Conditioned Medium: The Future for Regenerative Therapeutic Applications

**DOI:** 10.1155/2020/7593402

**Published:** 2020-01-31

**Authors:** Sara El Moshy, Israa Ahmed Radwan, Dina Rady, Marwa M. S. Abbass, Aiah A. El-Rashidy, Khadiga M. Sadek, Christof E. Dörfer, Karim M. Fawzy El-Sayed

**Affiliations:** ^1^Oral Biology Department, Faculty of Dentistry, Cairo University, Cairo, Egypt; ^2^Stem cells and Tissue Engineering Research Group, Faculty of Dentistry, Cairo University, Cairo, Egypt; ^3^Biomaterials Department, Faculty of Dentistry, Cairo University, Cairo, Egypt; ^4^Clinic for Conservative Dentistry and Periodontology, School of Dental Medicine, Christian Albrechts University, Kiel, Germany; ^5^Oral Medicine and Periodontology Department, Faculty of Dentistry, Cairo University, Cairo, Egypt

## Abstract

Regenerative medicine literature has proposed mesenchymal stem/progenitor cell- (MSC-) mediated therapeutic approaches for their great potential in managing various diseases and tissue defects. Dental MSCs represent promising alternatives to nondental MSCs, owing to their ease of harvesting with minimally invasive procedures. Their mechanism of action has been attributed to their cell-to-cell contacts as well as to the paracrine effect of their secreted factors, namely, secretome. In this context, dental MSC-derived secretome/conditioned medium could represent a unique cell-free regenerative and therapeutic approach, with fascinating advantages over parent cells. This article reviews the application of different populations of dental MSC secretome/conditioned medium in in vitro and in vivo animal models, highlights their significant implementation in treating different tissue' diseases, and clarifies the significant bioactive molecules involved in their regenerative potential. The analysis of these recent studies clearly indicate that dental MSCs' secretome/conditioned medium could be effective in treating neural injuries, for dental tissue regeneration, in repairing bone defects, and in managing cardiovascular diseases, diabetes mellitus, hepatic regeneration, and skin injuries, through regulating anti-inflammatory, antiapoptotic, angiogenic, osteogenic, and neurogenic mediators.

## 1. Introduction

Regenerative medicine employing tissue engineering approaches represents a promising emerging multidisciplinary branch of medicine that is aimed at regenerating as well as guiding restoration and enhancement of organs and tissues' functions, thereby improving the overall quality of life [1]. The goal remains to construct biological substitutes, mimicking the actual tissues and organs for therapeutic management of several diseases and disorders [2, 3]. In its course, this process requires combining biocompatible scaffolds, cells, proper signaling molecules, and physical stimuli [2, 4, 5].

Biocompatible scaffolds employed in tissue engineering, comprising a variety of natural, synthetic, conductive polymers, and elastic polymer networks such as hydrogels [6–8], combined with signaling molecules and/or growth factors [9–12]. In addition to polymers, scaffolds were further fabricated from bioceramics, bioactive glasses, and their composites [12–16]. In the same context, decellularization was introduced as a novel scaffold fabrication technique that depends on maintaining the extracellular matrix with its organization, architecture, and vascular network, thus obtaining a cell-free 3D structure harboring biological signals, affecting the cell behavior and differentiation [17]. Different methods were proposed for such decellularization process, including the employment of detergents, enzymes, and salts combined with some physical means [18], producing a biological scaffold, ready to be seeded by the desired cell type for different tissue engineering purposes [19, 20].

Different cell populations were proposed with remarkable properties to be used in the tissue engineering field, mainly adult stem/progenitor cells, embryonic stem cells, and induced pluripotent stem cells [21, 22]. Currently, adult mesenchymal stem/progenitor cells (MSCs) are among the most commonly investigated cells in tissue engineering endeavours. MSCs are multipotent cells, residing in numerous adult body tissues, including the bone marrow, adipose tissues, umbilical cord blood, and synovial fluid [23–25], hallmarked by their self-renewal abilities and differentiation potential into a multitude of cells of mesodermal origin, upon proper stimulation.

Although cellular transplantation of various MSCs has been proposed as a valid model for functional tissue regeneration, its translation into the clinical settings remains faced with various serious clinical obstacles. In recent years, MSCs have been characterized for their secretory ability of various bioactive molecules in their surrounding media (the conditioned media (CM)). These secreted molecules, also known as secretome, can be readily isolated, with demonstrated remarkable effects on mesenchymal tissue regeneration [26, 27]. Among the advantages reported for stem/progenitor cell-derived secretome over cell-based therapy are its ease of preservation, sterilization, packaging, and storage for extended periods without the risk of losing its properties. It can be accurately gauged for proper dosages and produced in large quantities, using cell lines without subjecting the patient to invasive extraction procedures, which is both time and cost saving [28–31]. In this review, we aim to investigate the efficacy of secretome derived from various dental mesenchymal stem/progenitor cell (dental MSC) populations in the therapeutic approaches of various diseases as well as on different tissues' regeneration, highlighting the bioactive molecules involved in their action.

## 2. Dental Stem/Progenitor Cells (Dental MSCs)

Dental MSCs are unique adult MSCs, derived from the ectomesenchyme's neural cells [32, 33]. They include dental pulp mesenchymal stem/progenitor cells (dental pulp MSCs) isolated from dental pulpal tissues of permanent teeth [34], stem/progenitor cells extracted from pulpal tissues of human shed deciduous teeth (SHED) [35, 36], periodontal ligament mesenchymal stem/progenitor cells (periodontal ligament MSCs) isolated from the periodontal ligament [37, 38], dental follicle mesenchymal stem/progenitor cells (dental follicle MSCs), usually isolated from the dental follicle surrounding the third molar [39], alveolar bone proper-derived mesenchymal stem/progenitor cells (alveolar bone MSCs) [40–42], mesenchymal stem/progenitor cells isolated from the apical dental papilla (MSCs from apical papilla) at the apices of the immature permanent teeth [38, 43], tooth germ progenitor cells, isolated from late bell stage third molar's tooth germs [44], and gingival mesenchymal stem/progenitor cells (gingival MSCs), isolated from gingival tissues [45–49]. Stem/progenitor cells have further been isolated from diseased dental tissues as inflamed pulp [50, 51] and periapical cysts [52, 53].

Dental MSCs express the common MSCs' surface markers, including CD105, CD73, and CD90 with a lack of expression of CD45, CD34, CD14, CD11b, CD79a, CD19, and human leukocyte antigen-DR isotype [54]. They are characterized by their ability to differentiate into multiple cell lineages, their self-renewal ability, their immunomodulatory properties, and their potent regenerative potentials [55–61]. Aside from their remarkable ease of acquisition via routine minimally invasive dental procedures [21], dental MSCs were reported to demonstrate an enhanced regenerative potential as compared to MSCs derived from other body tissues. Dental pulp MSCs [62–68], SHED [68], MSCs from the apical papilla [63–65], and dental follicle MSCs [63–65] revealed a higher osteogenic [63, 67], hepatogenic [64], neurogenic [65, 68], antiapoptotic [62], angiogenic [62, 69], pulpal tissue regenerative [62] potential and remarkable proliferative rates [70, 71] as compared to bone marrow-derived mesenchymal stem/progenitor cells (bone marrow MSCs) [62–68] or adipose stem/progenitor cells (adipose MSCs) [62, 66, 67].

## 3. Stem/Progenitor Cells' Secretome/Conditioned Medium

Apart from their direct cellular activity following stem/progenitor cells engraftment, the positive effect of stem/progenitor cells on target tissue repair and regeneration is indirectly mediated through paracrine effects [72–75]. The latter is mainly invoked through the release of trophic and modulatory bioactive factors (secretome) into the surrounding environment, by which they can influence tissue homeostasis and promote tissue regeneration [76, 77]. Secretome can induce cellular migration, proliferation, immunomodulation, and tissue regeneration [78–82]. Relying on this recently evolving concept, cell-free regenerative medicine approaches, utilizing stem/progenitor cells' secretome, have emerged as an alternative to cell-based therapies [73, 74, 83].

Secretome can be defined as the range of molecules secreted from living cells or shed from their surface into the extracellular environment [80]. Upon stimulation, stem/progenitor cells release secretome and trophic factors into the culture media, the stem/progenitor cells' CM [79, 84]. These stem/progenitor cells' secretome contains lipids, proteins, nucleic acid, and trophic factors as chemokines, cytokines, growth factors, hormones, and extracellular vesicles (EVs) [77]. Human cytokine array system, a useful tool for identifying novel cytokines [85], demonstrated that stem/progenitor cells derived from different anatomic locations show variation in secretome profile [86].

Regarding their composition, stem/progenitor cells' secretome was demonstrated to harbor an array of growth/differentiation factors, including vascular endothelial growth factor (VEGF), platelet-derived growth factor (PDGF), epidermal growth factor, insulin-like growth factor I and II (IGF-I, IGF-II), hepatocyte growth factor (HGF), fibroblast growth factor 2/basic fibroblast growth factor (FGF-2/bFGF), keratinocyte growth factor/fibroblast growth factor-7 (KGF/FGF-7), platelet-derived endothelial cell growth factor, heparin-binding epidermal growth factor, neural growth factor (NGF), and brain-derived neurotrophic factor (BDNF) [87]. Additionally, anti-inflammatory cytokines including transforming growth factor- (TGF-) *β*1 and interleukins (IL), including IL-6, IL-10, IL-27, IL-17, and IL-13, and proinflammatory cytokines including IL-8/CXCL-8, IL-9, and IL-1*β* were identified. Furthermore, granulocyte colony-stimulating factor (GCSF), granulocyte macrophage CSF (GM-CSF), and prostaglandin E2 (PGE2) were present [87].

### 3.1. Extracellular Vesicles (EVs)

EVs are secreted by many cell types, including stem/progenitor cells. They can be isolated from body fluids like urine, serum, and cerebrospinal fluids. Their content depends on the surrounding environment and may change upon cell stimulation. EVs include microvesicles (MVs) (100-1000 nm), exosomes (EXs) (40-100 nm), and apoptotic bodies (1-5 *μ*m) [80, 88–90]. Once EVs reach their target sites, they interact and attach to the target cell surface, where they either remain attached, become internalized by the target cell via fusion with the cell membrane as well as via the endocytotic pathway to discharge their content intracellularly, or become detached from the cell surface after completing their action [89, 91].

MVs and EXs are membrane-bound particles that are secreted by most cell types for normal homeostasis with their secretion increasing upon stimulation [91, 92]. Both MVs and EXs are pivotal for intercellular communication and can exert both paracrine and endocrine actions [91]. MVs and EXs can function as vehicles or stable transporters for the transfer of bioactive molecules as cytokines and growth factors from the producing cells to the adjacent or distant target cells through the circulation [89, 91, 92]. They can further deliver RNA to target cells to modify target cells' gene expression or protein synthesis [93, 94]. MVs and EXs differ in their cellular origin (biogenesis) as well as their physical characters, including size and surface markers [88, 95, 96]. Their content depends upon the producing cells, encompassing proteins and lipids, and protein-coding messenger RNAs and noncoding microRNA [90, 92, 96, 97].

MVs (also termed ectosomes) are heterogenous in size, ranging between 100 and 1000 nm in diameter. They are produced through direct budding from the cell plasma membrane, with their surface markers originating from the producing cells [95, 98]. MVs contain proteins and lipids, as well as mRNA and microRNA [99]. EXs, on the other hand, are homogenous and smaller in size with a diameter ranging from 40 to 100 nm. They originate in multivesicular bodies and are released from the cell through exocytosis via fusion with cell membrane [88, 100]. Following endocytosis, endocytotic vesicles are formed and fused giving rise to early endosomes that mature into late endosomes (multivesicular bodies), which eventually fuse with the membrane and discharge their content extracellularly [101]. EXs are rich in annexins, tetraspanins (CD63, CD81, and CD9), and heat-shock proteins (as Hsp60, Hsp70, and Hsp90), which are usually used for their identification [102].

### 3.2. Comparison between Secretome/Conditioned Media Derived from Dental MSCs and MSCs from Other Tissue Sources

A total of 1533 proteins were identified in the CM derived from bone marrow MSCs, adipose MSCs, and dental pulp MSCs by proteomic analysis. 999 proteins were contained in the CM of all three cell sources, of which 124 proteins were identified as secreted extracellular proteins. The secreted extracellular proteins were suggested to be responsible for the regenerative effects of MSCs including angiogenesis, migration, inflammatory response, ossification, and organ survival. A closer resemblance was notable between protein sets isolated from bone marrow MSC-CM and adipose MSC-CM rather than dental pulp MSC-CM [103]. Comparing MSCs from apical papilla-CM to bone marrow MSC-CM, proteins responsible for angiogenesis, immunomodulation, chemotaxis, neuroprotection, antiapoptosis, and extracellular matrix formation were detected in both CM. A significant difference in the levels of 151 of the detected proteins was however noticeable between the two cell sources, where MSCs from apical papilla-CM was associated with higher levels of proteins related to metabolic processes and transcription in addition to chemokines and neurotrophins and lower levels of proteins responsible for adhesion, immunomodulation, angiogenesis, and extracellular matrix proteins [104]. MSCs from the apical papilla-CM, dental follicle MSC-CM, and dental pulp MSC-CM showed a common expression of 174 cytokines. Dental pulp MSC-CM however revealed a significantly higher expression of 23 cytokines related to odontoblast differentiation, proinflammatory and anti-inflammatory cytokines, while three cytokines related to proliferation were significantly higher in MSCs from apical papilla-CM and dental follicle MSC-CM [105].

Regarding their tissue biological effects, dental pulp MSC-CM showed higher antiapoptotic, angiogenic, neurite outgrowth, migration activity [62, 106], and immunomodulatory effects in vitro as compared to bone marrow MSC-CM, in addition to higher vasculogenesis in vivo [106]. Dental pulp MSC-CM further demonstrated antiapoptotic effect and increased migration and angiogenesis on mouse embryonic muscle myoblast cells (C2C12) in vitro, which was attributed to the presence of high concentration of CXC motif ligand (CXCL14) and monocyte chemoattractant protein-1 (MCP-1) [107]. Dental MSC-CM derived from dental pulp MSCs, MSCs from the apical papilla, and dental follicle MSCs showed a superior nerve regenerative potential as compared to bone marrow MSC-CM, where dental MSC-CM were associated with significantly higher colony formation and neurite extension, indicating an enhanced neural differentiation and maturation, in comparison to bone marrow MSCs. This could be attributed to significantly higher levels of BDNF, neurotrophin-3 (NT-3) in dental MSC-CM derived from all three cell sources, and a significantly higher expression of NGF in MSCs from apical papilla-CM and dental follicle MSC-CM, as compared to bone marrow MSC-CM. Moreover, higher concentrations of GCSF, interferon gamma (IFN-*γ*), and TGF-*β* were detected in dental pulp MSC-CM as compared to bone marrow MSC-CM [65]. Similar results were notable, comparing the dental pulp MSC-CM to the bone marrow MSC-CM and adipose MSC-CM [66].

## 4. Stem/Progenitor Cells from Exfoliated Human Deciduous Tooth-Derived Secretome/Conditioned Medium (SHED-CM)

SHED, derived from the pulpal tissues of deciduous teeth, possess higher proliferation rate as compared to dental pulp MSCs and bone marrow MSCs. Microarray analysis showed that SHED had higher expression levels of FGF, TGF, connective tissue growth factor, NGF, and bone morphogenetic protein- (BMP-) 1 [108]. Gene encoding for extracellular, cell surface molecules, cell proliferation, and embryonic tissue development are highly expressed by SHED. Moreover, SHEDs expressed neural cell lineage markers including nestin, doublecortin, *β*-tubulin III, NeuN, glial fibrillary acidic protein (GFAP), S100, A2B5, and 2′,3′-cyclic-nucleotide 3′-phosphodiesterase [109]. In addition, SHED release an array of secretome with various biological therapeutic activities.

### 4.1. SHED-CM in the Therapy of Neural Injuries (Table 1)

SHED-CM contains various cytokines and chemokines with the ability to improve peripheral nerve regeneration and functional recovery [110]. The unique combination of neurotrophic factors, MCP-1 and secreted ectodomain of sialic acid-binding Ig-like lectin-9 (sSiglec-9), were described as crucial for SHED-CM mediated functional recovery, following severe peripheral nerve injury. This neuroprotective effect was evident through the promotion of migration, proliferation, and differentiation of Schwann cells; blood vessel formation; and nerve fiber extension [111]. These in vitro results were confirmed in vivo [110, 111]. SHED-CM administration in a rat nerve gap model induced axon regeneration and remyelination [110, 111]. Notably, MCP-1/sSiglec-9 prompted the polarization of M2 macrophages, which antagonized the proinflammatory M1 conditions associated with neural insult [111, 112], thereby increasing the expression of anti-inflammatory markers IL-10 and Arginine-1 and markedly suppressing inflammatory mediators IL-1*β*, tumor necrosis factor (TNF-*α*), IL-6, and inducible nitric-oxide synthase (iNOS) [111]. In a perinatal hypoxia-ischemia-induced brain injury mouse model, intracerebral administration of SHED-CM resulted in significant recovery in neurological function, survival rate, and neuropathological score [113]. The effects were primarily ascribed to the generation of an anti-inflammatory microenvironment, reducing tissue loss and thereby significantly improving the neurological outcome. In a further investigation, SHED-EXs reduced the proinflammatory microglia M1 phenotype cell markers in a dose-dependent manner and activated M2 microglia, thereby suppressing neuroinflammation by anti-inflammatory cytokines. These results were further proven in vivo [114, 115], where SHED-EXs improved rat motor functional recovery and reduced cortical lesion in a traumatic brain injury rat model [115]. Similarly, SHED-CM decreased infarct volume in contrast to bone marrow MSC transplantation in a focal cerebral ischemic study [114]. Moreover, SHED-CM promoted the migration and differentiation of endogenous neuronal progenitor cells, boosted vasculogenesis, and enhanced ischemic brain injury [114].

Both SHED-CM and dental pulp MSC-CM (as discussed below) significantly promoted transected axon regeneration, through inhibiting the multiple axon growth inhibitors signals directly or via paracrine mechanisms, as compared to fibroblast-CM or bone marrow MSC-CM. Moreover, the levels of MCP-1 and secreted ectodomain-Siglec-9 were higher in SHED-CM compared with bone marrow MSC-CM in vitro [109]. The neuroprotective effects were correspondingly confirmed in vivo [109, 112, 116], as SHED-CM improved functional recovery as compared with bone marrow MSC-CM [109, 112]. The therapeutic effect of SHED-CM was largely ascribed to immunoregulatory functions that activate anti-inflammatory M2-like macrophages and suppress proinflammatory mediators [112].

SHED-CM was further demonstrated to convert the proinflammatory brain/spinal cord environment to an anti-inflammatory state, through altering microglial phenotype as shown in a mouse model of Alzheimer's disease [117] and a mouse model of multiple sclerosis (MS) [118]. SHED-CM administration improved cognitive function more efficiently than the bone marrow MSC-CM or fibroblast-CM. SHED-CM, bone marrow MSC-CM, or fibroblast-CM similarly suppressed the proinflammatory cytokines and markers of oxidative-nitrosative stress expression. In contrast, SHED-CM uniquely activated M2-type microglia, which led to the expression of the mRNA encoding BDNF, a neurotrophin that plays an important role in the synaptic remodeling associated with memory formation. Interestingly, the same neuropathological recovery was observed in a previous study [113].

In an in vitro model of Parkinson's disease, SHED-CM demonstrated neuroprotective effects. SHED-CM enhanced neurite outgrowth and repressed 6-hydroxydopamine-induced cell death [119]. Similarly, SHED-CM showed a positive outcome in a Parkinson's disease rat model [120, 121]. A superior laryngeal nerve injury rat model was treated with systemic administration of SHED-CM and strikingly functional recovery was improved via two mechanisms: macrophage polarization and vascularization [122].

The previous data highlights the neural regenerative potential of SHED-CM that was primarily ascribed to the release of multiple growth factors, including NGF, BDNF, NT-3, ciliary neurotrophic factor, glial cell line-derived neurotrophic factor, and HGF [110], stimulation of angiogenesis by VEGF expression [123], and inhibition of 3-NT and iNOS generation [117]. Taken together, the results validated the potential of SHED-CM/EXs as a candidate for neuroprotective treatment of brain ischemia [114] and that SHED-CM may act through multiple mechanisms to provide neural functional recovery.

### 4.2. SHED-CM in the Therapy of Cardiopulmonary Injuries (Table 2)

SHED-CM induced the differentiation of mouse bone marrow-derived macrophages into M2 macrophages that expressed Arginase-1, Ym-1, and CD206 in vitro. These findings were further proved in vivo [124–126], where intravenous administration of SHED-CM in a bleomycin-induced acute lung injury mouse model, reduced lung fibrosis, and enhanced survival rates. These therapeutic effects were elicited through reducing the expression of proinflammatory cytokines and fibrotic markers such as *α*-smooth muscle actin, thereby reducing fibrosis by altering proinflammatory M1 into an anti-inflammatory M2 phenotype [112, 113, 126]. Furthermore, SHED-CM administration provided cardioprotective benefits in ischemic heart diseases, through at least two mechanisms, involving suppression of inflammatory responses in myocardial cells and reduction of cardiomyocyte death. These effects were greater compared to those of adipose SC-CM and bone marrow MSC-CM, owing to the significantly higher expression of HGF in SHED-CM as compared to the other two cell sources [127].

### 4.3. SHED-CM in the Therapy of Hepatic Disorders (Table 2)

Intravenous administration of SHED-CM in a liver failure mouse model exhibited a remarkable therapeutic effect that was not observed in the fibroblast-CM [124, 125]. TNF-*α*, IL-1*β*, and iNOS were strongly suppressed. Additionally, SHED-CM suppressed carbon tetrachloride-induced apoptosis in hepatocytes in vitro [124]. SHED-CM promoted anti-inflammatory cytokines (IL-10 and TGF-*β*1), M2 cell markers (CD206 and Arginase-1), angiogenic factor (VEGF) and hepatocyte proliferation, and antiapoptosis factor (stem cell factor and IGF-1) expression. Furthermore, SHED upregulated the expressions of LPC activation genes, including FGF 7, TWEAK, HGF, and Wnt3a [125]. These data suggest that the active biomolecules within the SHE-CM and endogenous tissue-repairing factors activated by the SHED-CM administration could function together to diminish liver failure-induced tissue destruction [124, 125].

### 4.4. SHED-CM in the Therapy of Diabetes Mellitus (Table 2)

The administration of the human SHED-CM and human bone marrow MSC-CM intravenously in a streptozotocin-induced diabetes model in rats resulted in the regeneration of pancreatic *β*-cells, with an increase in insulin secretion in the SHED-CM group. Moreover, the antidiabetic effect of SHED-CM was found to be superior to the bone marrow MSC-CM [128].

### 4.5. SHED-CM in the Therapy of Immunological Disorders (Table 2)

Human SHED-CM effect on rheumatoid arthritis was also investigated. SHED-CM or bone marrow MSC-CM injection intravenously in rats with induced arthritis demonstrated marked anti-inflammatory effects, a decrease in joint destruction and an overall improvement in arthritis symptoms, especially in the SHED-CM group. Additionally, SHED-CM inhibited osteoclastogenesis [129]. SHED-CM was further effective in suppressing inflammation and reducing inflammatory markers in chondrocytes cell culture treated with proinflammatory factors [130].

Similarly, human SHED-CM showed promising results in the treatment of alopecia in vivo and in vitro. In a study, mice with dorsal area shaved with clippers were injected subcutaneously with human SHED-CM or human hair follicle stem cell-CM. For the in vitro study, skin samples were obtained from the shaved dorsal skin of rats and cultured with CM. Results demonstrated that SHED-CM resulted in a faster stimulation of hair growth as compared to the hair follicle stem cell-CM, through upregulating positive hair growth-regulatory factors, stromal cell-derived factor-1, hair growth factor, VEGF-A, and PDGF-B [131].

### 4.6. SHED-CM in the Therapy of Dental Pulpal Disorders (Table 3)

The angiogenic effect of SHED-CM was studied on dental pulp in rats and on human umbilical vein endothelial cell culture (HUVECs). Endodontic treatment was performed on rats' first molar tooth followed by overinstrumentation with the last file to allow the blood clot to infill the root canal, and SHED-CM was applied on top of the blood clot. SHED-CM induced the formation of the vascular connective tissue inside the root canal. A similar inductive effect was observed in HUVEC cultures, indicating that SHED-CM has a proangiogenic effect in both in vitro and in vivo study models [123].

## 5. Dental Pulp Mesenchymal Stem/Progenitor Cell-Derived Secretome/Conditioned Medium

Dental pulp MSCs hold distinctive differentiation characteristics into ectodermal, endodermal, and the traditional mesodermal cell lineages [132]. In addition to MSC markers, dental pulp MSCs express neural stem cell-like markers, including nestin and GFAP, which are believed to amplify their multipotency and self-renewal abilities [133]. Remarkably, dental pulp MSCs express stemness-related markers as Oct-3/4, Nanog, and sex-determining region Y- (SRY-) box 2 (SOX-2) [134], in addition to a variety of angiogenic factors such as VEGF, PDGF, and FGF, with an interesting increase of their expression after injury [135], as well as CSF, IL-8, angiogenin, endothelin-1, angiopoietin-1, and IGF-binding protein-3 [136–138]. Dental pulp MSCs demonstrate immunomodulatory properties partly attributable to their expression of IL-8, IL-6, and TGF-*β*, which could inhibit T cell function [139, 140]. Moreover, dental pulp MSCs secrete many neurotrophic factors like BDNF [141], glial cell line-derived neurotrophic factor [142], and NGF [143].

Although dental pulp MSCs and SHED originate from dental pulpal tissues and share many common properties, SHED demonstrated a higher proliferation rate but lower osteogenic potential as compared to dental pulp MSCs [144]. On the other hand, the proliferative potential and telomerase activity of dental pulp MSCs were higher than periodontal ligament MSCs [145]. The aforementioned properties of dental pulp MSCs hallmark their distinctiveness, which is further reflected into the remarkable therapeutic paracrine effect of their secretome/CM.

### 5.1. Dental Pulp MSC-CM in the Therapy of Neural Disorders (Table 1)

Similar to SHED-CM, dental pulp MSC-CM demonstrated remarkable neural regenerative potentials, with the ability to induce recruitment, neuronal maturation, and neuritogenesis of human neuroblastoma cells in vitro [146], in addition to neurite outgrowth [106]. The regenerative effect of dental pulp MSC-CM, bone marrow MSC-CM, and adipose MSC-CM were compared in an in vitro model of retinal nerve damage. Dental pulp MSC-CM demonstrated neuroprotection and neuritogenesis attributed to their increased levels of different neurotrophic factors, including NGF, BDNF, and VEGF [66]. Moreover, dental pulp MSC-CM promoted proliferation, differentiation, and migration of Schwann cells and inhibited their apoptosis, as well as enhanced angiogenesis in an in vitro model of nerve injury [147]. Dental pulp MSC-CM further revealed a neuroprotective effect in an in vitro model of Alzheimer's disease. Their effect was attributed to the increase in the expression of B-cell lymphoma 2 and the decrease in apoptosis regulator Bax in neuroblastoma cells. Moreover, dental pulp MSC-CM contains a high concentration of neprilysin, which cause the degradation of amyloid-*β* peptide (one of the major misfolded protein accumulated in Alzheimer's disease), fractalkine (antiapoptotic factor), and VEGF compared to bone marrow MSC-CM or adipose MSC-CM, in addition to RANTES, FLT-3, GM-CSF, and MCP-1, which make them a promising candidate in treating Alzheimer's disease [148]. Dental pulp MSC-CM also provided a neuroprotective effect in an in vitro model of hypoxic ischemic brain damage. Dental pulp MSC-CM showed an increase in cell viability and a decrease in cell apoptosis in comparison with bone marrow MSC-CM. Moreover, dental pulp MSC-CM provided an increase in the number and total length of tubular structures of HUVECs in an in vitro ischemia model [69].

The therapeutic potential of dental pulp MSC-CM systemic administration in a mutant superoxide dismutase mouse model of amyotrophic lateral sclerosis was demonstrated [149]. Dental pulp MSC-CM improved neuromuscular junction innervation and motor neuron survival in treating amyotrophic lateral sclerosis through different trophic factors and cytokines [149]. Similarly, dental pulp MSC-CM exhibited neuroprotective, anti-inflammatory, and angiogenic actions when administrated into unilateral hind limb skeletal muscles of a diabetic polyneuropathy rat model [150]. Intrathecal administration of dental pulp MSC-CM in a rat aneurysmal subarachnoid hemorrhage model revealed improvement in cognitive and motor impairments, microcirculation, and reduction of neuroinflammation. IGF-1, TGF-*β*, tissue inhibitor of metalloproteinase- (TIMP-) 1, and TIMP-2 were identified as significant components in dental pulp MSC-CM that contribute to these improvements [151].

Collectively, these data clearly demonstrated that dental pulp MSC-CM harbors an array of neuroprotective and angiogenic factors such as NGF, BDNF and VEGF [66], RANTES, fractalkine, FLT-3, GM-CSF, MCP-1, and neprilysin [148], besides IGF-1, TGF-*β*, TIMP-1, and TIMP-2 [151], which account for their promising abilities to induce tissue regeneration in many neurological diseases.

### 5.2. Dental Pulp MSC-CM Osteogenic Potential (Table 4)

The surrounding microenvironment could impact on the osteogenic differentiation of dental pulp MSCs [152]. Dental pulp MSCs cultured with dental pulp MSC-CM demonstrated an enhanced mineralization potential [153]. In a further study evaluating the regenerative potential of dental pulp MSC-CM grown under different culture conditions in a distraction osteogenesis mouse model, dental pulp MSC-CM increased osteoblastic and chondrogenic markers' expression, with accelerated bone healing especially in CM collected under hypoxic conditions [154]. These findings indicate that the paracrine influence of dental pulp MSCs could initiate new bone formation through increasing the mineralization potential by expressing TGF-*β*1 [153], in addition to upregulating angiogenic factors (VEGF-A and angiopoietin-2), as well as enhancing osteoblastic and chondrogenic marker expression (osterix, SOX-5, and factor VIII) [154].

### 5.3. Dental Pulp MSC-CM in the Therapy of Hepatic Disorders (Table 2)

Another promising regenerative application of dental pulp MSC-CM was demonstrated in the field of hepatic therapy. Dental pulp MSC-CM remarkably demonstrated the presence of various hepatic lineage proteins, including hepatocyte nuclear factor, growth arrest specific-protein, oncostatin M, and hepatocyte growth factor receptor in vitro [64], thereby promoting hepatic repair and regeneration.

### 5.4. Dental Pulp MSC-CM in Dental Tissue Regeneration (Table 3)

EXs derived from dental pulp MSCs demonstrated a potent stimulatory effect on odontoblastic differentiation in vitro and triggered regeneration of dental pulp-like tissue in vivo in an ectopic tooth transplantation model [155]. Dental pulp MSC-CM enhanced the proliferation and migration of the myoblast [156] and fibroblast [157] in vitro, which was confirmed in vivo in an ectopic tooth transplantation model [107]. The addition of G-CSF to CM from mobilized dental pulp MSCs [157] improved the proliferation and migration effect of dental pulp MSC-CM. Dental pulp MSC-CM promoted dental pulp MSC differentiation into odontoblasts in vitro [62]. These results could be attributed to high concentrations of NT-3 or BMP in dental pulp MSC-CM [105]. On the other hand, dental pulp MSC-CM alone failed to induce odontoblastic differentiation in cells of nondental origin like myoblast [156]. The regenerated tissues by dental pulp MSC-CM demonstrated the expression of pulp tissue markers including syndecan 3, thyrotropin-releasing hormone-degrading enzyme, CXCL14, G-CSF, BDNF, neuropeptide Y, IL-1*α*, IL-6, IL-8, IL-16, MCP-1 [107], BMP2, BMP9, TGF-*β*, PDGF, runt-related transcription factor 2 (RUNX2), and dentin sialophosphoprotein [155] in addition to enamelysin as well as periodontal tissue markers, including periodontal ligament-associated protein (PLAP-1) and periostin [156].

Several studies were carried out comparing the regenerative capacity of dental pulp MSC-CM to that of other cell sources. Pulp regeneration was assessed using an ectopic tooth model seeded with bone marrow MSC-CM, adipose MSC-CM, and dental pulp MSC-CM. Dental pulp MSC-CM showed the highest volume of regenerated pulp tissues as compared to CM from other cell sources. Dental pulp MSC-CM showed angiogenic effect in an in vitro pulp disease model of HUVECs [62, 156] and embryonic muscle myoblast cells [107] as well as antiapoptotic activity on mouse embryonic fibroblast cell line (NIH3T3) [106]. Dental pulp MSC-CM promoted neovascularization as compared with bone marrow MSC-CM and adipose MSC-CM [107]. Dental pulp MSC-CM had no significant effect on the proliferation of endothelial cells but enhanced their migration in vitro [138]. Moreover, dental pulp MSC-CM inhibited apoptosis in HUVECs [158] and fibroblast cell line through modulating caspase-3 activity [157]. Various angiogenic factors were identified in dental pulp MSC-CM such as VEGF, IGF-binding protein 3, IL-8, endostatin [138], MCP-1 [107, 138], and chemokine CXCL 14 [107]. The aforementioned studies highlight dental pulp MSC-CM as a new promising therapeutic tool for dental tissue regeneration through different mechanisms of action, including promoting odontoblastic differentiation, angiogenesis, and antiapoptotic factors. Exploring their therapeutic potential in nondental tissue regeneration will be of a great benefit.

## 6. Gingival Mesenchymal Stem/Progenitor Cell-Derived Secretome/Conditioned Medium

Gingival MSCs are a subpopulation of MSCs that could be isolated from the lamina propria of gingival connective tissues [49, 159, 160], with remarkable regenerative properties [161, 162]. Compared to other MSCs, gingival MSCs are abundant, homogenous, and easily obtainable with faster proliferation rate [48]. Gingival MSCs preserve normal karyotyping and maintain stable morphology in later passages as compared to bone marrow MSCs, with remarkable multidirectional differentiation potential and immune regulatory properties [48, 160, 163–166]. In addition to MSC surface markers, gingival MSCs express CD13, CD38, CD44, CD54, CD117, CD144, CD146, CD166, Sca-1, STRO-1, SSEA-4, Oct-3/4, Oct-4A, Nanog, nestin, integrin *β*1, and vimentin [49, 159, 167]. In addition, gingival MSCs could release an array of secretome with various biological therapeutic actions.

### 6.1. Gingival MSC-CM in the Therapy of Neural Disorders (Table 1)

Various investigations suggested that gingival MSC-derived EXs, EVs, or CM could represent novel therapeutic interventions in managing peripheral nerve injury [168, 169], motor neuron injury [170], and skin [171] and bone defects [172]. The results were comparable with effects conferred by direct transplantation of gingival MSCs [168, 169]. The regenerative effect of EXs derived from human gingival MSCs combined with biodegradable chitin conduits on peripheral nerve injury was investigated. Gingival MSC-EXs significantly promoted the in vitro proliferation of Schwann cells as well as the growth of a DRG axon. In vivo assessment of the repair of a 10 mm defect of the sciatic nerve in rats revealed a significant increase in the thickness of nerve fibers and the myelin sheath. Besides, the muscle and neuromuscular functions were recovered [169]. In an in vitro study, the gingival MSCs derived EVs embedded on locally wrapping gel-foam proved to exert beneficial effects on the functional recovery and axonal repair/regeneration of the crush-injured sciatic nerve in mice. The gingival MSC-EVs robustly upregulated the expression of several repair Schwann cell-related genes c-JUN, Notch1, GFAP, and SOX-2, significantly blocking the activity of c-JUN/N-terminal kinase (c-JUN/JNK), which normally abolishes the upregulation of Schwann cell repair genes [168]. The neuroprotective capability of human gingival MSC-CM on scratch-injured motor-neuron-like NSC-34 cells was evolved by suppressing apoptotic markers (cleaved caspase-3 and Bax), oxidative stress markers (superoxide dismutase- (SOD-) 1, iNOS), while upregulating anti-inflammatory cytokine (IL-10) and neurotrophic factor (BDNF and NT-3) expressions. In addition, NGF, NT-3, IL-10, and TGF-*β* were detected in human gingival MSC-CM [170].

In critical-sized tongue defect model in rats, involving the combinative transplantation of small intestinal submucosa-extracellular matrix with gingival MSCs or their derivative, EXs proved to regenerate tongue lingual papillae and taste buds, with an increasing expression of CK14^+^ (basal epithelial progenitor cells' marker); CK8^+^ (intragemmal cells' marker); type I, II, and III taste bud cells' markers (NTPdase 2, PLC-*β*2, and AADC, respectively), in addition to nerve fiber markers (UCH-L1/PGP9.5 and P2X_3_ receptor). Moreover, the expression of two key trophic factors (BDNF and Shh), with remarkable roles in the proliferation and differentiation of basal epithelial progenitor cells into taste bud cells and the reconstruction of submucosal connective tissues [173], was promoted. The faster wound healing rate in the gingiva was primarily attributed to the gingival MSCs and their unique secretory mechanism through the Fas/Fas-associated phosphatase-1 (Fap-1)/caveolin-1 (Cav-1) complex that triggers SNARE-mediated membrane fusion to secrete a large quantity of IL-1 receptor antagonist- (IL-1RA-) expressing EVs, inhibiting the proinflammatory cytokine IL-1*β* [174]. This finding represents an auspicious application potential for tongue reconstruction in patients suffering from tongue cancer. All these studies propose gingival MSCs' secretome/CM as a simple and autologous therapeutic tool to repair/regenerate nerve injuries, mainly through increasing the expression of anti-inflammatory cytokines (IL-10), antiapoptotic cytokine (Bcl2) [170], and markers denoting neural growth (BDGF, NT-3, Neurofilament 200, S100) [168–170, 173], as well as enhancing proliferation and regeneration of nerve cells detected by PCNA [168], CCK-8 [169], and Shh [173] aside from a suppression of proinflammatory cytokine TNF-*α* [170], IL-17, IFN-*γ* [175, 176], and proapoptotic (Bax and cleaved caspase-3) and oxidative stress markers (SOD-1, iNOS, COX-2) [170].

### 6.2. Gingival MSC-CM in the Therapy of Skin Injuries (Table 2)

The implementation of gingival MSC-derived EXs in skin repair proved to be of practical value. Isolated EXs with an average diameter of 127 nm derived from gingival MSCs loaded on chitosan/silk hydrogel sponge effectively promoted healing of skin defects in diabetic rats detected by the formation of neoepithelium and collagen as well as a rise in the microvessels' number detected by CD34 in the wound bed and neuronal ingrowth detected by neurofilament heavy chain (NEFH), two weeks postsurgery [171].

### 6.3. Gingival MSC-CM Osteogenic Potential (Table 4)

In bone regenerative medicine, the osteogenic regenerative potential of a poly-(lactide) (3D-PLA) scaffold supplemented with human gingival MSCs and human gingival MSC-CM was explored in rat calvaria bone defects, demonstrating a marked increase in bone contact after six weeks. Moreover, in vitro next-generation sequencing confirmed the increase in the genes involved in ossification (ASF1A, GDF5, HDAC7, ID3, INTU, PDLIM7, PEX7, RHOA, RPL38, SFRP1, SIX2, SMAD1, SNAI1, SOX-9, and TMEM64) in the 3D-PLA loaded with the gingival MSC-CM group [172]. This was basically attributed to the growth factors and cytokines contained in the CM that could activate mobilization and osteogenic differentiation of both endogenous MSCs and gingival MSCs [28–31, 172]. In a further study, EVs derived from human gingival MSCs were complexed with polyethyleneimine (PEI) to improve their internalization and performance. The PEI-engineered EVs were similarly loaded on 3D-PLA combined with human gingival MSCs. In vitro, the 3D-PLA+PEI-EVs+human gingival MSCs demonstrated greater osteogenic capabilities as emphasized by more calcium depositions six weeks later. In the 3D-PLA+PEI-EVs+human gingival MSCs construct, transcriptomic analysis demonstrated an upregulation of 31 genes involved in ossification processes as well as 21 genes involved in the regulation of adhesion molecules. Also, in vivo computed tomography (CT) revealed the formation of new bone spicules and blood vessels in rats' calvarial bone defects implanted with 3D-PLA+PEI-EVs+human gingival MSCs and 3D-PLA+PEI-EVs. It was hypothesized that the osteogenic potential of PEI-EV-human gingival MSCs loaded on 3D-PLA was mediated mainly by TGF-*β*R1, SMAD1, BMP2, MAPK1, MAPK14, and RUNX2 through TGF-*β* signaling [177].

Hence, harvesting human gingival MSCs and their secretome/CM is easy and harmless to the patients and relatively inexpensive. The previous findings provide a promise for their utilization in bone tissue engineering, especially in the repair of cranial bone defects.

## 7. Periodontal Ligaments Mesenchymal Stem/Progenitor Cell-Derived Secretome/Conditioned Medium

The periodontal ligament is considered a potent source of stem/progenitor cells for tissue regeneration that can differentiate into several types of cells [178–180]. They are the most favorable stem/progenitor cell population utilized in periodontal regeneration [181], due to their high expression of scleraxis, a protein responsible for the formation of the cementum-periodontal ligament complex [37]. Human periodontal ligament MSCs are similar to bone marrow MSCs, with high proliferative rate, immunomodulatory functions, and an in vitro differentiation ability into osteogenic, adipogenic, chondrogenic, and neurogenic cell lineages [182–184]. Periodontal ligament MSCs express proteins that are not present in bone marrow MSCs including CLPP, NQO1, SCOT1, a new isoform of TBB5, and DDAH1, explaining the unique properties of periodontal ligament MSCs [185–187].

Similar to other MSCs, the therapeutic effects of human periodontal ligament MSCs and their key role in alveolar bone and periodontal ligament homeostasis could be mediated through secreted paracrine signaling molecules [175]. Human periodontal ligament MSCs were demonstrated to regulate the osteogenic and adipogenic differentiation of alveolar bone MSCs and inhibit alveolar bone MSC-induced osteoclastogenic differentiation of human peripheral blood mononuclear cells [188]. Additionally, periodontal ligament cell-CM can modulate the expression of genes responsible for cell proliferation and bone homeostasis from MSCs upon coculturing with BMP-2 [189].

The analysis of the cytokine profile of permanent and deciduous periodontal ligament cells revealed that proteins concerned with immune responses and degradation were detected more strongly in deciduous periodontal ligament-CM, while cytokines related to angiogenesis (epidermal growth factor and IGF-1) and neurogenesis (NT-3 and NT-4) were contained in permanent periodontal ligament-CM making them a potential candidate for tissue regeneration [190]. Moreover, the cytokine analysis of epithelial cell rests of Malassez, harbored within the periodontal ligament, revealed the expression of significant amounts of chemokines; growth factors and related proteins as IL-1, IL-6, IL-8, and IL-10; GM-CSF; MCP-1, 2, and 3; amphiregulin, glial cell line-derived neurotrophic factor, and VEGF and IGF-binding protein 2 [191].

### 7.1. Periodontal Ligament MSC-CM in the Therapy of Neural Disorders (Table 1)

The immunosuppressive effects of human periodontal ligament MSCs and their secretome in managing multiple sclerosis were investigated [175, 192]. In vitro characterization of human periodontal ligament MSC-CM showed an increased level of IL-10, TGF-*β*, and stromal cell-derived factor 1*α* [175]. In an in vivo study, the reverse in disease progression and remyelination of the spinal cord in an experimental autoimmune encephalomyelitis model was assigned to the EXs/MVs (EMVs) fractions of human periodontal ligament MSC-CM. Periodontal ligament MSC-CM and periodontal ligament MSC-EMVs reduced proinflammatory cytokines TNF-*α*, IL-17, IL-6, IL-1*β*, and IFN-*γ* and induced anti-inflammatory IL-10 expression, as well as attenuated the expression of apoptosis-related markers Bax, STAT1, caspase-3, and p53 in the spleen and spinal cord [175]. In a more recent study, downregulated expressions of NALP3 inflammasome, cleaved caspase-1, IL-1*β*, IL-18, Toll-like receptor- (TLR-) 4, and nuclear factor- (NF-) *κ*B were demonstrated in an experimental autoimmune encephalomyelitis mouse spinal cord after treatment with human periodontal ligament MSC-CM and EMVs. Finally, it was concluded that both human periodontal ligament MSC-CM and purified EMVs exerted comparable immunosuppressive effects and that CM alone may serve as an effective and economical therapeutic tool in multiple sclerosis treatment [192].

Similarly, the ability of human periodontal ligament MSC-CM under hypoxic condition to repress induced experimental autoimmune encephalomyelitis in a murine model was studied [176]. Hypoxic human periodontal ligament MSC-CM was injected through the tail vein of the mice. The clinical and histological features of the disease were diminished accompanied by a marked expression of anti-inflammatory and antiapoptotic (cytokine IL-37 and protein Bcl-2, respectively) as well as suppression of pro-inflammatory and pro-apoptotic markers (cleaved caspase-3 and Bax, respectively). Moreover, treating the in vitro scratch injury model-exposed neurons NSC-34 via hypoxic-human periodontal ligament MSC-CM demonstrated therapeutic action [176]. The aforementioned studies propose periodontal ligament MSC-CM as a new pharmacologic tool for managing multiple sclerosis through a remarked expression of anti-inflammatory cytokines (IL-10, TGF-*β*) [175, 176] and antiapoptotic cytokine (Bcl2) [170, 176] and subsequent suppression of proinflammatory mediators (IL-4, IL-17, IFN-*γ*, TNF-*α*, IL-6, and IL-1*β*) [175, 176], proapoptotic markers (Bax and cleaved caspase-3) [170, 175, 176], p53, STAT1 [175], cleaved caspase-1 [192], and oxidative stress markers (SOD-1, iNOS, and COX-2) [170, 176]. A reduction in the NALP3, IL-1*β*, IL-18, TLR-4, and NF-*κ*B expressions were reported to mediate the nerve regenerative effect of periodontal ligament MSCs [192]. Moreover, periodontal ligament MSC-CM upregulated expression of markers denoting neural growth such as IL-37, BDNF, and NT-3, besides markers of autophagy (Beclin-1, LC3) [176].

### 7.2. Periodontal Ligament MSC-CM Osteogenic Potential (Table 4)

Bone regeneration and angiogenic potential of a 3D collagen membrane (3D-COL) loaded with human periodontal ligament MSCs and CM or EVs or EVs treated with PEI (PEI-EVs) in calvarial defects in rats were studied. In vitro results demonstrated an initially increased expression of osteogenic markers (RUNX2 and BMP-2/4) in human periodontal ligament MSCs cultured within the 3D-COL and PEI-EVs, associated with increased protein levels of VEGF, VEGF receptor-2 (VEGFR-2), and collagen type 1. The increased expression of these proteins was confirmed in clavarial defects implanted with the 3D-COL loaded with human periodontal ligament MSCs and PEI-EVs. Moreover, histological examination and micro-CT imaging confirmed this regenerative ability [193]. Likewise, Evolution (Evo) (a commercially available collagen membrane) loaded with human periodontal ligament MSCs enriched with EVs and PEI-EVs demonstrated high biocompatibility and osteogenic properties in vitro and in rats' calvarial defects. A quantitative reverse-transcription polymerase chain reaction showed upregulation of osteogenic genes MMP-8, TGF-*β*1, TGF-*β*2, tuftelin-interacting protein (TFIP11), tuftelin 1 (TUFT1), RUNX2, SOX-9, and BMP2/4 in the presence of PEI-EVs. The increased expression of BMP-2/4 was confirmed for the collagen membrane loaded with PEI-EVs and human periodontal ligament MSCs both in vitro by Western blot and in vivo by immunofluorescence [194]. Ultimately, these results demonstrated that human periodontal ligament MSCs might be an effectual strategy in bone regenerative medicine, consequent to its potential to increase osteogenic and angiogenic mediators through the TGF-*β*-BMP signaling pathway.

### 7.3. Periodontal Ligament MSC-CM in Dental Tissue Regeneration (Table 3)

In treating periodontal tissue defects, transplanted periodontal ligament MSC-CM as compared to fibroblast-CM was investigated in a rat periodontal defect model. Periodontal ligament MSC-CM transplantation enhanced periodontal tissue regeneration via suppressing the inflammatory response induced by TNF-*α*, IL-6, IL-1*β*, and COX-2. Proteomic analysis revealed that extracellular matrix proteins, angiogenic factors, enzymes, growth factors, and cytokines were contained in periodontal ligament MSC-CM [181].

## 8. Dental Follicle Mesenchymal Stem/Progenitor Cells (Dental Follicle MSCs), Mesenchymal Stem/Progenitor Cells from the Apical Papilla (MSCs from the Apical Papilla) and Tooth Germ Progenitor Cell-Derived Secretome/Conditioned Medium (Tables 1, 2, and 4)

Expressing Nestin, Notch1, collagen type I, bone sialoprotein, osteocalcin, and fibroblast growth factor receptor 1-IIIC [39], dental follicle MSCs demonstrated osteogenic and cementogenic differentiation capacity in vitro and in vivo [39, 195, 196]. Similar to dental follicle MSCs, MSCs from the apical papilla possess odontogenic and adipogenic differentiation ability [43, 197] and express neurogenic markers in vitro without induction [197]. Being the primary source of odontoblasts at root region, MSCs from the apical papilla have the ability to differentiate into dentin-pulp complex [198]. MSCs from apical papilla and dental follicle MSCs revealed comparable hepatogenic differentiation potential and superior neurogenic ability to bone marrow MSCs [65, 169].

The regenerative potential of human dental pulp MSC-CM, human dental follicle MSC-CM, and human MSCs from apical papilla-CM in nerve [65], liver [64], and bone regeneration [63] was investigated in vitro. Human dental pulp MSC-CM, human dental follicle MSC-CM, human MSCs from apical papilla-CM, and human bone marrow MSC-CM were collected and cultured with preneuroblast cell line IMR-32. Dental MSC secretome stimulated colony formation in IMR-32 and neurite differentiation with a significant increase in neural gene expression (MFI, MAP-2, *β*-tubulin III, nestin, and SOX-1) more efficiently as compared with bone marrow MSCs' secretome. Moreover, the dental MSCs' secretome showed a significantly higher expression of growth factors and cytokines involved in neural regeneration (CSF, IFN-*γ*, TGF-*β*, NGF, NT-3, and BDNF) as compared to bone marrow MSCs. On the contrary, IL-17 expression was higher in bone marrow MSC-CM as compared to dental pulp MSC-CM [65].

Dental MSC-CM could further provide a valuable tool for liver regeneration. The presence of hepatic lineage proteins GAS6 in the secretome of dental pulp MSCs, MSCs from the apical papilla, and dental follicle MSCs and different LDL receptor (LRP) proteins in the secretome of dental pulp MSCs and MSCs from the apical papilla reflected their role in controlling lipid metabolism and transport as well as hepatic differentiation. Interestingly, oncostatin M and hepatocyte growth factor receptor, which are important inducers for hepatic lineage differentiation were detected solely in dental follicle MSC secretome [64].

The presence of osteogenic lineage proteins was demonstrated in high amounts in human dental pulp MSC-CM which contained seven proteins, including BMP7 and dentin sialophosphoprotein; human dental follicle MSC-CM which contained six proteins, including proteins regulating endochondral ossification (MINPP1), bone turnover (WISP2) and mineralization (enamelin); and human MSCs from apical papilla-CM, which contained 14 proteins including four of the five proteins detected in bone marrow MSC-CM, among them, FBN1, DDR2, and Zinc finger protein- (ZNF-) 423 that play important roles in osteoblastic maturation, activation of BMPs, and differentiation of bone osteocytes, respectively [63]. The expression of these osteogenic proteins could open numerous possibilities for applications of dental MSC-CM in the regeneration of bone disorders.

## 9. Dental MSC-CM Biological Effects

### 9.1. Immunomodulatory and Anti-inflammatory Effects

It is well known that the immunomodulatory and anti-inflammatory effects of MSC-CM are mediated through soluble immune-regulatory molecules. Dental MSC-CM induce an immunoregulatory activity by converting the proinflammatory conditions and induced anti-inflammatory M2-like macrophage differentiation, thereby treating neural diseases [111, 112, 115, 117, 118, 122], lung injury [126], and liver failure [125]. Dental MSC-CM promoted anti-inflammatory cytokines (IL-10 and TGF-*β*1) as well as M2 cell markers (CD206 and Arginase-1) [125]. Moreover, dental MSC-CM suppressed the expression of the proinflammatory cytokines TNF-*α* [170, 175, 176, 181]; IL-4, IL-17 and IFN-*γ* [175, 176]; IL-6 and IL-1*β* [175, 176, 181]; COX-2 [181]; and NALP3, IL-18, TLR-4, and NF-*κ*B [192]. The balance between these anti-inflammatory and proinflammatory cytokines may determine the final effect.

### 9.2. Neuroprotective and Neurotrophic Effects

Although neurodegenerative diseases and other neural insults represent a major challenge as they currently do not have an effective treatment, dental MSC-CM opened the way for treating these challenging conditions. Several studies supported the neuroregenerative effects of dental MSC-CM [66, 69, 109, 110, 112, 116, 168–170, 173]. The key role of dental MSC-CM as a modulator of the neurogenic microenvironment is through the release of multiple growth factors promoting neural growth and differentiation like NGF [65, 66, 110, 117]; BDNF [65, 66, 110, 117, 168–170, 173, 176]; NT-3 [65, 110, 168–170, 173, 176]; CNTF, GDNF, and HGF [110]; IGF [117, 151]; MFI, MAP-2, *β*-tubulin III, nestin, and SOX-1 [65], besides Neurofilament 200 and S100 [168–170, 173]. Moreover, dental MSC-CM contained factors involved in the reduction of neurotoxicity such as VEGF, RANTES, FRACTALKINE, FLT-3, and MCP-1 and A*β*-degrading enzyme neprilysin [148]. All these factors combined or in isolation act to ameliorate and treat the neural diseases.

### 9.3. Osteogenesis

Dental MSC-CM promotes osteogenesis through enhancing the migration and mineralization potential of MSCs by TGF-*β*1 [153] as well as the upregulation of their osteoblastic and chondrogenic marker expression (Osterix, SOX-5, factor 8) [154]. In this context, TGF-*β*-BMP signaling pathway plays a pivot role in osseous regeneration induced by dental MSCs and their secretome through upregulating the expression of TGF-*β*1, TGF-*β*2, BMP2, BMP4, MMP8, TUFT1, TFIP11, RUNX2, and SOX-9 was detected [194], as well as VEGF, VEGFR2, and COL1A1 [193]. The osteoblastic differentiation potential is primarily mediated by TGF-*β*R1, SMAD1, BMP2, MAPK1, MAPK14, and RUNX2 through the TGF-*β* signaling pathway [177]. Interestingly, 15 genes involved in the ossification process were only detected in dental MSC-CM [172]. Dental MSC-CM contained BMP7 and DSPP that play a key role in bone formation and mineralization as well as protein regulating, endochondral ossification (MINPP1), bone turnover (WISP2), mineralization (enamelin) and FBN1, DDR2, and ZNF423 that play important roles in osteoblastic maturation, activation of BMPs, and differentiation of bone osteocytes, respectively [63]. Thus, dental MSC-CM possesses pivotal biomolecules to greatly promote the cellular osteogenic potential.

### 9.4. Hepatic Regeneration

Currently, in terminal stages of fibrosis, liver transplantation is the only effective treatment modality. Yet, due to the accompanying clinical obstacles, including low supply of suitable donors and transplant rejection, the development of therapeutic approaches for liver fibrosis are seriously required [124]. The described therapeutic effect of dental MSC-CM in liver fibrosis is primarily ascribed to numerous factors involved in antiapoptosis/hepatocyte protection (SCF and IGF-1), angiogenesis (VEGF), macrophage differentiation, and the proliferation/differentiation of hepatic lineage and LPCs including OSM and HGFR [64, 125].

### 9.5. Angiogenic Effect

The molecular and cellular events of angiogenesis are tightly controlled by a delicate balance between stimulatory and inhibitory signals. Dental MSC-CM promoted angiogenesis through the secretion of proangiogenic factors including VEGF-A, angiopoietin-2, MMP3, G-CSF GM-CSF, G-CSF, IL-8, MCP-1, uPA, TIMP-1, and PAI-1, aside from endogenous angiogenesis inhibitors (IGFBP-3 and endostatin). These factors play important roles in promoting hair growth [131], new bone formation [154], and dental tissue regeneration [138, 158].

### 9.6. Anti-Apoptotic Effect

Dental MSC-CM may provide substantial therapeutic benefits through its antiapoptotic action via the release of antiapoptotic markers that increase cell survival, including Bax and cleaved caspase-3 [170, 175, 176]; p53 and STAT1 [175]; cleaved caspase-1 [192]; SCF; and IGF-1 [125] and through modulating caspase-3 activity [157].

### 9.7. Modulation of Oxidative Stresses

A delicate balance normally exists between antioxidants and oxidants in human tissues, where excessive reactive oxygen species (ROS) are effectively neutralized by antioxidants [199, 200]. Low concentrations of ROS could be beneficial, aside from oxidation-reduction (redox) reactions, which may have a regulatory function, in protecting cells from apoptosis [201]. On the contrary, higher concentrations of ROS may cause direct cells' oxidation, aggravated inflammation, unregulated autophagy activity, and drives apoptosis, eventually resulting in tissue damage and dysfunction [199, 200, 202]. Based on such phenomenon, oxidative stress-related diseases such as burning mouth syndrome could be effectively treated by antioxidants [203].

Among the major challenges concerning the clinical application of MSCs is to maintain their genetic integrity [204, 205] against ROS that generate DNA damage in vitro, resulting in an oxidative modification of DNA bases or spontaneous hydrolysis of nucleosides [206]. Adipose MSC-CM incubation with human dental fibroblast cells helped these cells to release antioxidant enzymes and resist oxidative free radicals [207]. Additionally, treating mouse ischemic/perfused hearts and utilizing MSC-derived EXs increased ATP and NADH levels and decreased oxidative stress [208].

The ability of dental MSC-CM to modulate oxidative stresses has further been proposed as a possible therapeutic mechanism. It has been reported that periodontal ligament MSC-CM [176] and gingival MSC-CM [170] significantly reduced markers of oxidative stresses as SOD-1, iNOS, and COX-2, in an in vitro model of multiple sclerosis and together with their immunomodulatory and antiapoptotic properties significantly reduced neural cells' death [170, 176]. Similarly, dental pulp MSC-CM and SHED-CM effectively reduced ROS production in neural cells [209], a mouse model of Alzheimer's disease [117] and multiple sclerosis [118]. In treating periodontal defects, periodontal ligament MSC-CM suppressed COX-2 levels, suggesting a close relationship between periodontal ligament MSC-CM transplantation, reduction in inflammation, and periodontal tissue regeneration [181].

## 10. Conclusion

Dental MSC-derived secretome holds a multitude of capacities for tissue engineering and regenerative medicine. Utilizing stem/progenitor cells secretome in regenerative medicine is further considered advantageous and can overcome limitations associated with stem cell-based therapies. Following transplantation, stem/progenitor cells demonstrate a low survival rate [210] and a potential risk of malignant transformation, particularly subsequent to their in vitro expansion to acquire the adequate number of cells for clinical use [73, 211]. A cell-free secretome/CM therapeutic strategy could restore back the function of damaged tissues via the activation of signaling pathways based on the transfer of bioactive molecules, proteins, and mRNAs to the affected tissues. Such a therapy could avoid the risks of tumorgenicity, antigenicity, host rejection, and infection associated with stem cell-based therapies, constituting a safer and more convenient source for regenerative bioactive molecules as compared to stem/progenitor cells engraftment.

Dental MSC secretome/CM demonstrate numerous advantages. In accordance with their origin, dental MSC secretome/CM expresses significantly higher levels of cytokines related to odontoblastic differentiation. Compared to nodental MSC secretome/CM, dental MSC secretome/CM demonstrate higher levels of metabolic, transcriptional, and proliferation-related proteins, chemokines, and neurotrophins, while lower levels of proteins responsible for adhesion and extracellular matrix production. Dental MSC secretome/CM show higher antiapoptotic, angiogenic, neurite outgrowth, migration activity, vasculogensis, and immunomodulatory effects. They further demonstrate superior nerve regenerative, differentiation, and maturation potentials, with significantly higher colony formation and neurite extension.

Even though stem cell secretome has many potential applications in tissue regeneration, several issues should be addressed to facilitate its translation into clinical trials. Developing a manufacturing protocol compliant with good manufacturing practice, without using any animal-based products, in addition to determining the exact dosage, frequency of administration, exact protein composition, and mechanism of action are a must before carrying secretome application into human patients. With accumulating technology and experience, the clinical applications of dental MSC secretome still warrant further research to explore the full potentials of dental MSCs' secretome in the regeneration of different oral and extra oral tissues.

## Figures and Tables

**Table 1:** Summary of the included studies investigating the effect of dental MSCs' secretome/conditioned medium on neurogenic regeneration.

Authors, year	Cell origin-contributing factor	Scaffold	Study model	Factors contained in dental MSC-CM	Factors promoted by dental MSC-CM	Outcome
Neural regeneration and treating neural disorders						
SHED-CM						
Sakai et al., 2012 [109]	Human SHED-CM Human dental pulp MSC-CM	-	In vivo spinal cord injury. In vitro.	-	-	Promoted neural regeneration.
Inoue et al., 2013 [114]	Human SHED-CM	-	In vivo rat with cerebral ischemia.	DCX, NF, NeuN, & RECA1.	-	Promoted neuronal progenitor cells migration, differentiation, and vasculogenesis.
Yamagata et al., 2013 [113]	Human SHED-CM	-	In vivo hypoxic ischemic brain injury mouse.	IL-1*β* & TNF-*α*.	-	Improved neurological function, inhibited apoptosis, and decreased tissue loss.
Fujii et al., 2015 [119]	Human SHED-CM	-	In vivo Parkinson's disease model. In vitro	-	-	Promoted neurite outgrowth of neurons and inhibited neuron apoptosis.
Jarmalaviciute et al., 2015 [120]	Human SHED-EXs and MVs	-	In vitro Parkinson's disease.	-	-	Stimulated neurite outgrowth of neurons and inhibited neuron apoptosis.
Matsubara et al., 2015 [112]	Human SHED-CM	-	In vivo rat with spinal cord injury.	M2 markers (IL-10, CD206) & M2-like macrophage inducers: MCP-1, Siglec-9, & IL-6.	-	Regenerated neurons suppressed inflammation which promoted functional recovery.
Mita et al., 2015 [117]	Human SHED-CM	-	In vivo Alzheimer's disease. In vitro.	Ym-1, Arginase-1, & Fizz1. IL-10, mRNA of BDNF, NGF, & IGF.	-	Protected against neurodegeneration, improved cognitive functions, and inhibited neuroblastoma cell apoptosis.
Sugimura-Wakayama et al., 2015 [110]	Human SHED-CM	-	In vivo sciatic nerve defect. In vitro	NGF, BDNF, NT-3, GDNF, CNTF, VEGF, & HGF.	NGF, BDNF, NT-3, CNTF, GDNF, VEGF, laminin, fibronectin, & collagen type IV.	Promoted axon regeneration, remyelination, and motor functional recovery. Increased Schwann cell proliferation, migration, and activation.
Shimojima et al., 2016 [118]	Human SHED-CM	-	In vivo multiple sclerosis mouse model.	ED–Siglec-9 & HGF.	mRNAs of Arginase-1 & CD206. ↓ mRNA of iNOS.	Reduced axon injury, demyelination, and reduced inflammation.
Kano et al., 2017 [111]	Human SHED-CM	Collagen sponge	In vitro & in vivo peripheral nerve injury.	MCP-1 & sSiglec-9.	mRNAs of Arginase-1, Cd206, & Il-10.	Mediated neurological regeneration. Schwann cell proliferation, migration, and differentiation.
Li et al., 2017 [115]	Human SHED-EXs	-	In vivo rat with traumatic brain injury. In vitro	CD9, CD63, & CD81.	↓ TNF-*α*, IL-6, CD11b, CD68, mRNA of CD11b, CD86, CD16, MHCII, iNOS, CD206, IL-10, & Arginase-1.	Improved motor functional recovery and reduced neuroinflammation.
Asadi-Golshan et al., 2018 [116]	Human SHED-CM	Collagen hydrogel	In vivo rat spinal cord injury.	-	-	Enhanced neurological functional recovery.
Tsuruta et al., 2018 [122]	Human SHED-CM	-	In vivo superior laryngeal nerve injury dysphagia in rat.		Arginase-1, IL-10, Lif, Ccl2, NGF, BDNF, NTN, and mRNA VEGF. ↓ iNOS & IL-1*β*.	Promoted axonal regeneration and enhanced angiogenesis.
Narbute et al., 2019 [121]	Human SHED-EVs	-	In vivo rat with Parkinson's disease.	-	-	Suppression of gait impairments and normalization of tyrosine hydroxylase expression.
Dental pulp MSC-CM						
Ishizaka et al., 2013 [106]	Porcine dental pulp MSC-CM	-	In vitro	-	-	Triggered antiapoptotic activity on fibroblast and promoted neurite outgrowth of human neuroblastoma cell line.
Mead et al., 2014 [66]	Human dental pulp MSC-CM	-	In vitro retinal nerve damage.	NGF, BDNF, & VEGF.	-	Showed the presence of different neurotrophic factors.
Ahmed et al., 2016 [148]	Human dental pulp MSC-CM	-	In vitro Alzheimer's disease.	VEGF, RANTES, fractalkine, FLT-3, GM-CSF, MCP-1, & neprilysin.	Bcl-2 & Bax.	Inhibited apoptosis in neuroblastoma cell line and increased its viability.
Yamamoto et al., 2016 [147]	Human dental pulp MSC-CM	-	In vitro nerve section.	-	-	Induced proliferation, differentiation, and migration of Schwann cells and inhibited their apoptosis.
Gervois et al., 2017 [146]	Human dental pulp MSC-CM	-	In vitro	-	-	Induced recruitment, neuronal maturation, and neuritogenesis of human neuroblastoma cells.
Song et al., 2017 [69]	Human dental pulp MSC-CM	Endothelial cell medium gel	In vitro model of ischemia.	-	-	Increased the number and total length of tubular structures in HUVECs.
Chen et al., 2019 [151]	Rat dental pulp MSC-CM	-	In vivo rat with aneurysmal subarachnoid hemorrhage.	IGF-1, TGF-*β*, TIMP1, & 2.	-	Improvement of microcirculation and neuroinflammation.
Makino et al., 2019 [150]	Rat dental pulp MSC-CM	-	In vivo rat with diabetic polyneuropathy. In vitro	-	-	Exhibited neuroprotective, anti-inflammatory, and angiogenic actions. Increased proliferation of HUVEC in vitro.
Wang et al., 2019 [149]	Human dental pulp MSC-CM	-	In vivo mouse with amyotrophic lateral sclerosis.	-	-	Improved neuromuscular junction innervation and motor neuron survival.
Gingival MSC-CM						
Rajan et al., 2017 [170]	Human gingival MSC-CM	-	In vitro neuron degenerative diseases.	NGF, NT-3, IL-10, & TGF-*β*.	Bcl-2, IL-10, BDNF, & NT-3. ↓ SOD-1, iNOS, COX-2; TNF-*α*, cleaved caspase-3, & Bax.	Suppression of neural cell apoptosis, oxidative stress, and inflammation.
Mao et al., 2019 [168]	Human gingival MSC-EVs Human gingival MSC-CM	-	In vivo mouse with sciatic nerve injury. In vitro	-	Postsynaptic AChR clusters in NMJ, *β*-tubulin III, S100*β*, GFAP, c-JUN, Notch1, SOX-2, EGR2/KROX-20, PCNA, BrdU.	Promoted proliferation, migration of Schwann cells, axonal regeneration, and functional recovery.
Rao et al., 2019 [169]	Human gingival MSC-EXs	-	In vivo rat with sciatic nerve injury. In vitro	-	Neurofilament 200, S100, & CCK8.	Promoted increase in number of nerve fibers, myelin formation, recovery of muscle and nerve function, Schwann cell proliferation, and cell axon growth.
Zhang et al., 2019 [173]	Human gingival MSC-EXs	SIS-ECM	In vivo critical-sized tongue defect in rats.	-	CK14, CK8, NTPdase 2, PLC-*β*2, AADC, UCH-L1/PGP9.5, BDNF, P2X_3_, & Shh.	Promoted tongue lingual papillae recovery and taste bud regeneration and re-innervation.
Periodontal ligament MSC-CM						
Rajan et al., 2016 [175]	Multiple sclerosis human periodontal ligament MSC-CM Multiple sclerosis human periodontal ligament MSC-EVMs Human periodontal ligament MSC-CM Human periodontal ligament MSC-EVMs.	-	In vivo mouse with multiple sclerosis.	-	IL-10, TGF-*β* ↓ IL-4, IL-17, IFN-*γ*, TNF-*α*, IL-6, IL-1*β*, STAT1, p53, caspase-3, & Bax.	Promoted anti-inflammatory, immunosuppressive effects and downregulated apoptosis-related genes.
Giacoppo et al., 2017 [176]	Hypoxia—human periodontal ligament MSC-CM	-	In vivo mouse with multiple sclerosis. In vitro	NT-3, IL-10, & TGF-*β*	IL-37, caspase-1, IL-10, BDNF, NT-3, Bcl-2; Beclin-1, LC3; phosphorylation of PI3K, Akt, & mTOR. ↓ IL-17, IFN-*γ*, JNK, TNF-*α*, iNOS, COX-2, cleaved caspase- 3, & Bax.	Clinical and histologic features of the disease were diminished via modulation of inflammation, oxidative stress, and apoptotic pathways.
Rajan et al., 2017 [192]	Multiple sclerosis human periodontal ligament MSC-CM Multiple sclerosis human periodontal ligament MSC-EMVs	-	In vivo mouse with multiple sclerosis.	Substantial level of IL-10, TGF-*β*, & SDF-1*α* Less amount of IL-15, MCP-1, and MIP-1*α*.	↓ NALP3, cleaved caspase-1, IL-1*β*, IL-18, TLR-4, & NF-*κ*B.	Promoted anti-inflammatory and immunosuppressive effects.
Dental follicle MSC-CM & MSCs from apical papilla-CM						
Kumar et al., 2017 [65]	Human dental pulp MSC-CM Human dental follicle MSC-CM Human MSCs from apical papilla-CM	-	In vitro	GM-CSF, IFN-*γ*, TGF-*β*, NGF, BDNF, NT-3	MFI, MAP-2, *β*-tubulin III, nestin, and SOX-1	Enhanced neural differentiation.

AADC: aromatic l-amino acid decarboxylas; AChR: acetylcholine receptor; Akt: protein kinase B; Bax: Bcl-2-associated X protein; Bcl-2: B-cell lymphoma 2; BDNF: brain-derived neurotrophic factor; BrdU: bromodeoxyuridine; CCK8: Cell Count Kit-8; Ccl2: chemokine C-C motif ligand; CD: cluster of differentiation; CM: conditioned medium; CNTF: ciliary neurotrophic factor; COX-2: cyclooxygenase 2; DCX: doublecortin; ED-Siglec-9: ectodomain of sialic acid-binding Ig-like lectin-9; EGR2/KROX: early growth response gene; EVs: extracellular vesicles; EXs: exosomes; Fizz 1: resistin-like molecule alpha 1; FLT-3: Fms-related tyrosine kinase 3; GDN: glial cell line-derived neurotrophic factor; GFAP: glial fibrillary acidic protein; GM-CSF: granulocyte-macrophage colony-stimulating factor; MSCs: mesenchymal stem cells; HGF: hepatocyte growth factor; HUVECs: human umbilical vascular endothelial cells; IGF: insulin-like growth factor; IL: interleukin; iNOS: inducible nitric oxide synthase; JNK: c-Jun N terminal kinases; Lif: leukemia inhibitory factor; MAP-2: microtubule associated protein 2; MCP-1: monocyte chemoattractant protein-1; MHC: major histocompatibility complex; MIP-1*α*: macrophage inflammatory protein-1*α*; mTOR: mammalian target of rapamycin; MVs: microvesicles; NALP3: NACHT domain-, leucine-rich repeat-, and PYD-containing protein 3; NeuN: hexaribonucleotide binding protein 3; NF: nuclear factor; NF-*κ*B: nuclear factor, kappa light chain enhancer of activated B-cells; NGF: nerve growth factor; Notch 1: neurogenic locus notch homolog protein; NT-3: neurotrophin 3; NTN: neurturin; NTPdase 2: ectonucleotidases; P2X_3_: purinergic receptor P2X_3_; p53: tumor protein p53; PCNA: proliferating cell nuclear antigen; MSCs: stem cells; PI3K: phosphoinositide 3- kinases; PLC-*β*2: phospholipase c *β*2; RANTES: chemokine (c-c motif) ligand 5 (CCL5); RECA1: homolog of bacteria RecA; S100*β*: S 100 calcium-binding protein *β*; SDF-1*α*: stromal cell-derived factor 1*α*; Shh: sonic hedgehog; Siglrc-9: sialic acid-binding immunoglobulin type lectins-9; SOD-1: superoxide dismutase; SOX: sex-determining region Y-box; STAT1: signal transducer and activator of transcription 1; TLR: Toll-like receptor; TNF: tumor necrosis factor; UCH-L1/PGP9.5: ubiquitin carboxyterminal hydrolase isozyme 1; VEGF: vascular endothelial growth factor.

**Table 2:** Summary of the included studies investigating the effect of dental MSCs' secretome/conditioned medium on treating skin and internal diseases.

Authors, year	Cell origin-contributing factor	Scaffold	Study model	Factors contained in dental MSC-CM	Factors promoted by dental MSC-C	Outcome
Treating cardiopulmonary injuries						
SHED-CM						
Wakayama et al., 2015 [126]	Human SHED-CM	-	In vivo mouse with acute lung injury.	-	CD206, Arginase-1, & Ym-1	Suppressed inflammatory chronic response of macrophage and promoted lung regeneration.
Yamaguchi et al., 2015 [127]	Human SHED-CM	-	In vivo mouse with ischemia-reperfusion.	VEGF, IGF-1, HGF, bFGF, SDF-1, EGF, & SCF.	↓ TNF-*α*, IL-6, & IL-1*β*.	Reduced the size of myocardial infarct, myocyte apoptosis and inflammatory cytokine.
Diabetes mellitus						
SHED-CM						
Izumoto-Akita et al., 2015 [128]	Human SHED-CM	-	In vivo diabetic mouse model. In vitro	-	-	Increased insulin secretion, *β*-cell proliferation, and reduced apoptosis.
Immunological disorders						
SHED-CM						
Ishikawa et al., 2016 [129]	Human SHED-CM	-	In vivo model of rheumatoid arthritis.	HGF, IL-22, furin, IL-1RA, RAGE, OPG, MCP-1, & ED-Siglec-9.	RANKL, TRAP, Cathepsin K, RANK, NFATc1, OPG, CD206, Arginase-1, & Fizz1.	Promoted M2 anti-inflammatory state and inhibited osteoclastogenesis.
Gunawardena et al., 2019 [131]	Human SHED-CM	-	In vivo mouse model of alopecia. In vitro	-	SDF-1, HGF, VEGF-A, PDGF-BB, IL-1*α*, IL-1*β*, TNF-*α*, TGF-*β*, bFGF, & BDNF.	Stimulation of hair growth.
Luo et al., 2019 [130]	Human SHED-EXs	-	In vitro TMJ osteoarthritis model.	CD9, CD63, TSG101, & MiR-100.	↓ IL-6, IL-8, MMP1, MMP3, MMP9, MMP13, ADAMTS5, MMP1, MMP9, MMP13, & mTOR.	Suppression of inflammation in TMJ osteoarthritis.
Treating skin injuries						
Gingival MSC-CM						
Shi et al., 2017 [171]	Human gingival MSC-EXs	Hydrogel	In vivo diabetic rat with skin defect.	-	CD34, Neurofilament 200	Improved skin healing via reepithelialization, collagen deposition, enhanced angiogenesis and neuronal ingrowth.
Hepatic regenerative potential						
Dental pulp MSC-CM, dental follicle MSC-CM, & MSCs from apical papilla-CM						
Hirata et al., 2016 [124]	Human SHED-CM	-	In vivo mouse with liver fibrosis.	HGF	mRNA of MMP13, ↓ collagen type 1 (a1 and a2), & *α*-smooth muscle actin mRNAs.	Inhibited chronic inflammation and hepatocytes apoptosis.
Matsushita et al., 2017 [125]	Human SHED-CM	-	In vivo rat with acute liver failure.	HGF, MMP-10, MCP-1, ANG, SCF, IGFBP-2, sIL-6R, EGFR, FSTN, MMP-3, spg130, GRO, MIP-1*β*, MIF, RAGE, TIMP-4, adipsin, OPG, CXCL16, IGFBP-1, BDNF, LAP, GDNF, sTNFR1, TGF-*β*2, FGF-7, MMP-13, MMP-9, Flt-3 L, Dkk-3, NID-1, VEGF-A, CTSS, HVEM, GDF-15, TIMP-1, B2M, EG-VEGF, *β*-IG-H3, TIMP-2, IL-6, MCP-3, PAI-1, uPAR, IGFBP-6, Dkk-1, MMP-1.	IL-10, TGF-*β*1, CD206, Arginase-1, VEGF, SCF, and IGF-1, FGF 7, TWEAK, HGF, & Wnt3a genes.	Enhanced the condition of the injured liver and induced anti-inflammatory M2-like hepatic macrophages.
Kumar et al., 2017 [64]	Human dental pulp MSC-CM	-	In vitro	LRP6, LRP10, LRP5, LRP4, GAS6.	-	Demonstrated the presence of hepatic lineage proteins.
	Human dental follicle MSC-CM			APC, PEG10, GAS6, OSM, HGFR.		
	Human MSCs from apical papilla-CM			APC, ABCB4, APOA, GAS6, LRP4, LRP1, LRP1B, LRP8, LRP3, LRP4, APOC3, HNF4G.		
	Human bone marrow MSC-CM			APC, PEG10, ABCB4, APOBR, APOA, LPA.		

ABCB4: phosphatidylcholine translocator; Adipsin: complement factor D; ANG: angiogenin; APC: adenomatous polyposis coli protein; APOA: apolipoprotein A; APOBR: apolipoprotein B receptor; APOC3: apolipoprotein C-III; B2M: *β*2-microglobulin; BDNF: brain-derived neurotrophic factor; bFGF: basic fibroblast growth factor; CD: cluster of differentiation; CM: conditioned medium; CTSS: cathepsin S; CXCL16; chemokine (C–X–C motif) ligand 16; Dkk-1: Dickkopf 1; Dkk-3: Dickkopf 3; ED-Siglec-9: ectodomain of sialic acid-binding Ig-like lectin-9; EGF: epidermal growth factor; EGFR: epithelial growth factor receptor; EG-VEGF: endocrine-gland-derived vascular endothelial growth factor; EXs: exosomes; FGF: fibroblast growth factor; Flt-3 L: Fms-like tyrosine kinase receptor-3; FSTN: follistatin; GAS6: growth arrest-specific protein 6; GDF-15: growth differentiation factor 15; GDNF: glial cell line-derived neurotrophic factor; GRO: chemokine (C–X–C motif) ligand 1; HGF: hepatocyte growth factor; HGFR: hepatocyte growth factor receptor; HNF4G: hepatocyte nuclear factor 4 gamma; HVEM: herpesvirus entry mediator; IGF-1: insulin-like growth factor 1; IGFBP: insulin-like growth factor binding protein; IL: interleukin; IL-1RA: interleukin-1 receptor antagonist; LAP: latency-associated peptide; LPA: lipoprotein A; LRP10: LDL receptor-related protein 10; LRP1B: low-density lipoprotein-related protein 1B; LRP3: LDL receptor-related protein 3; LRP4: LDL receptor-related protein 4; LRP5: LDL receptor-related protein 5; LRP6: LDL receptor-related protein 6; LRP8: LDL receptor-related protein 8; MCP-1: monocyte chemoattractant protein-1; MCP-3: monocyte chemoattractant protein-3; MIF: macrophage migration inhibitory factor; MIP-1*β*: macrophage inflammatory protein 1*β*; MMP: matrix metalloprotease; mRNA: messenger RNA; MSCs: mesenchymal stem cells; mTOR: mammalian target of rapamycin; NFATc1: nuclear factor of activated T cells 1; NID-1: nidogen-1; OPG: osteoprotegerin; OSM: oncostatin M; PAI-1: plasminogen activator inhibitor-1; PDGF: platelet-derived growth factor; PEG10: retrotransposon-derived protein; RAGE: receptor for AGEs; RANK: receptor activator of nuclear factor-*κ*B; RANKL: receptor activator of nuclear factor-*κ*B ligand; SCF: stem cell factor; SDF-1: stromal cell-derived factor 1; MSCs: mesenchymal stem cells; SHED: stem cells derived from human exfoliated deciduous teeth; sIL-6R: soluble interleukin-6 receptor; spg130: soluble glycoprotein 130; sTNFR1: soluble tumor necrosis factor receptor 1; TGF-*β*: transforming growth factor-*β*; TIMP: tissue inhibitor of metalloproteinases; TNF-*α*: tumor necrosis factor alpha; TRAP: tartrate-resistant acid phosphatase; TWEAK: TNF-related weak inducer of apoptosis; uPAR: urokinase plasminogen activator surface receptor; VEGF: vascular endothelial growth factor.

**Table 3:** Summary of the included studies investigating the effect of dental MSCs' secretome/conditioned medium on dental and periodontal tissue regeneration.

Authors, year	Cell origin-contributing factor	Scaffold	Study model	Factors contained in dental MSC-CM	Factors promoted by dental MSC-CM	Outcome
Dental tissue regeneration						
SHED-CM						
de Cara et al., 2019 [123]	Human SHED-CM	-	In vivo orthotropic model of dental pulp regeneration in rats. In vitro	-	VEGF-A &↓ 7AAD	Stimulated angiogenesis, formation of connective tissue similar to dental pulp, and reduced apoptosis.
Dental pulp MSC-CM						
Iohara et al., 2008 [158]	Porcine dental pulp MSC-CM	-	In vitro	-	MMP3, VEGF-A, GM-CSF, & G-CSF.	Promoted macrovascular proliferation of HUVECs and inhibited its apoptosis.
Bronckaers et al., 2013 [138]	Human dental pulp MSC-CM	-	In vitro	VEGF, IL-8, MCP-1, uPA, TIMP-1, PAI-1, IGFBP-3, & endostatin.	FGF-2	Enhanced endothelial cell migration and blood vessels formation.
Hayashi et al., 2015 [107]	Porcine dental pulp MSC-CM	Root with collagen.	In vivo ectopic tooth transplantation mouse model.	TRH-DE mRNA.	Syndecan 3, TRH-DE, CXCL14, G-CSF, BDNF, NPY, IL-1*α*, IL-6, IL-8, IL-16, and MCP-1.	Promoted odontoblastic migration, proliferation, differentiation, and neovascularization.
Murakami et al., 2015 [62]	Dog dental pulp MSC-CM	-	In vitro pulp disease.	-	DSPP & enamelysin.	Induced dental pulp MSC proliferation, migration, and odontoblastic differentiation. Stimulated HUVECs angiogenesis.
Huang et al., 2016 [155]	Human dental pulp MSC-EXs	Type I collagen membranes and root slice. Collagen sponges.	In vivo ectopic tooth transplantation. In vitro	-	BMP2, BMP9, TGF-*β*, PDGF, RUNX2, & DSPP.	Stimulated dental pulp MSCs odontoblastic differentiation.
Kawamura et al., 2016 [156]	Porcine dental pulp MSC-CM	Root -	In vivo ectopic tooth transplantation mouse model. In vitro pulp disease.	-	TRH-DE, enamelysin, PLAP-1, & periostin. Vascular endothelial cadherin.	Promoted myoblasts proliferation, migration, and odontoblastic differentiation in the presence of EDTA. Stimulated HUVECs angiogenesis.
Nakayama et al., 2017 [157]	Human dental pulp MSC-CM	-	In vitro	-	↓ caspase-3	Mobilized dental pulp MSC-CM promoted fibroblast proliferation and migration, and inhibited its apoptosis.
Periodontal tissue regeneration						
Periodontal ligament MSC-CM						
Nagata et al., 2017 [181]	Human periodontal ligament MSC-CM	-	In vivo rat with periodontal defect	TIMP1, uPA, VEGF, IGFBP6, IGFBP2, PDGF-*β*, collagen, fibronectin & less amount of Serpin E1, MCP-1.	↓ TNF-*α*, IL-6, IL-1*β*, & COX-2.	Promoted new tissue formation and periodontal tissue healing.

BDNF: brain-derived neurotrophic factor; BMP: bone morphogenetic protein; CM: conditioned medium; COX-2: cyclooxygenase-2; CXCL14: chemokine (C-X-C motif) ligand 14; DSPP: dentin sialophosphoprotein; EXs: exosomes; FGF: fibroblast growth factor; G-CSF: granulocyte colony-stimulating factor; GM-CSF: granulocyte-macrophage colony-stimulating factor; HUVECs: human umbilical vascular endothelial cells; IGFBP: insulin-like growth factor-binding protein; IL: interleukin; MCP-1: monocyte chemoattractant protein-1; MMP: matrix metalloproteinase; mRNA: messenger RNA; MSCs: mesenchymal stem cells; NPY: neuropeptide Y; PAI-1: plasminogen activator inhibitor-1; PDGF: platelet-derived growth factor; PLAP-1: periodontal ligament-associated protein 1; RUNX2: runt-related transcription factor 2; Serpin E1: serine protease inhibitor E1; SHED: stem cells derived from human exfoliated deciduous teeth; TGF-*β*: transforming growth factor-*β*; TIMP-1: tissue inhibitor of metalloproteinase-1; TNF-*α*: tumor necrosis factor alpha; TRH-DE: thyrotropin-releasing hormone degrading enzyme; uPA: urokinase plasminogen activator; VEGF: vascular endothelial growth factor.

**Table 4:** Summary of the included studies investigating the effect of dental MSCs' secretome/conditioned medium on bone regeneration.

Authors, year	Cell origin-contributing factor	Scaffold	Study model	Factors contained in dental MSC-CM	Factors promoted by dental MSC-CM	Outcome
Bone regeneration						
Dental pulp MSC-CM						
Paschalidis et al., 2014 [153]	Human dental pulp MSC-CM	-	In vitro	-	TGF-*β*1	Enhanced dental pulp MSCs viability, migration and mineralization potential.
Fujio et al., 2017 [154]	Human dental pulp MSC-CM	-	In vivo mouse with distraction osteogenesis. In vitro	VEGF-A & angiopoietin-2	Osterix, SOX-5, & factor 8.	Hypoxic dental pulp MSC-CM enhanced angiogenesis and increased osteoblastic and chondrogenic markers expression.
Gingival MSC-CM						
Diomede et al., 2018 [172]	Human gingival MSCs+ Human gingival MSC-CM	PLA	In vivo rat calvarial defect. In vitro	ASF1A, GDF5, HDAC7, ID3, INTU, PDLIM7, PEX7, RHOA, RPL38, SFRP1, SIX2, SMAD1, SNAI1, SOX-9, BCAP29, BMP2K, DHRS3, FAM20C, TMEM64, FHL2, & TOB2.		Induction of new bone formation and osseointegration through expressing or upregulating genes involved in ossification or regulation of ossification.
Diomede et al., 2018 [177]	Human gingival MSCs+ Human gingival MSC-EVs Human gingival MSCs+ PEI- Human gingival MSC-EVs	PLA	In vivo rat calvarial defect. In vitro	FHL2, BMP2, TWSG1, CCDC47, FAM20C, ERCC2, LEP, TOB2, IMPAD1, CHRDL1, MINPP1, HIRA, MYBBP1A, JAG1, MEF2C, SUCO, SFRP1, SOX-9, SIX2, RHOA, PDLIM7, IFT80, SMAD1, HDAC7, ASF1A, ID3, SNAI1, PEX7, RPL38, BMP2K, and BCAP29.	RUNX2 & BMP2/4.	Improved bone healing by showing better osteogenic properties and exhibiting greater osteogenic inductivity.
Periodontal ligament MSC-CM						
Diomede et al., 2018 [194]	Human periodontal ligament MSCs+ Human periodontal ligament MSC-EVs or human periodontal ligament MSC-PEI-EVs	Collagen membrane	In vivo rat calvarial defect In vitro	-	TGF-B1, TGF-B2, BMP2, BMP4 MMP8, TUFT1, TFIP11, RUNX2 SOX-9.	Increased osteogenic potential and enhanced osseous regeneration and osseointegration processes.
Pizzicannella et al., 2019 [193]	Human periodontal ligament MSCs+ Human periodontal ligament MSC-CM or human periodontal ligament MSC-EVs or human periodontal ligament MSC-PEI-EVs	3D collagen membrane	In vivo rat calvarial defect In vitro	-	VEGF, VEGFR2, RUNX2, COL1A1, BMP2, & BMP4.	Enhanced osseous regeneration, vascularization, and osseointegration.
Dental follicle MSC-CM & MSCs from apical papilla-CM						
Kumar et al., 2018 [63]	Human dental pulp MSC-CM	-	In vitro	SAMD9, ADAM19, BMP7, ATP2B4, DSPP, BEST3, & LRP4.	-	Revealed the presence of osteogenic lineage proteins important for osteogenic differentiation.
	Human dental follicle MSC-CM			ATP2B4, MINPP1, ENAM, WISP2, COL27A, & ITGB3.	-	
	Human MSCs from apical papilla-CM			FBN1, DDR2, ZNF423, SAMD9, ADAM19, BMP7, ATP2B4, USP9X, ZNF521, INHBA, ROR2, LRP4, COL27A, & ITGB3.	-	
	Human bone marrow MSC-CM			FBN1, BMPR1A, DDR2, ZNF423, SAMD9.	-	

3D: three dimensional; ADAM19: disintegrin and metalloproteinase domain-containing protein; ASF1A: anti-silencing function 1A histone chaperone; ATP2B4: plasma membrane calcium transporting ATPase 4; BCAP29: B-cell receptor-associated protein 29; BEST3: bestrophin-3; BMP: bone morphogenetic protein; BMP2K: BMP2-inducible kinase; BMPR1A: bone morphogenetic protein receptor type-1A; CCDC47: coiled-coil domain containing 47; CHRDL1: chordin-like 1; CM: conditioned medium; COL1A1: collagen type 1; COL27A1: collagen alpha-1(XXVII) chain; DDR2: discoidin domain receptor family, member 2; DSPP: dentin sialophosphoprotein; ENAM: enamelin; ERCC2: ERCC excision repair 2; TFIIH: core complex helicase subunit; EVs: extracellular vesicles; FAM20C: Golgi-associated secretory pathway kinase; FBN1: fibrillin 1; FHL2: four and a half LIM domains 2; GDF-5: growth differentiation factor 5; HDAC7: histone deacetylase 7; HIRA: histone cell cycle regulator; ID3: inhibitor of DNA binding 3; IFT80: intraflagellar transport 80; IMPAD1: inositol monophosphatase domain containing 1; INHBA: inhibin beta A chain inhibitor-1; ITGB3: integrin beta-3; JAG1: jagged 1; LEP: leptin; LRP4: LDL receptor-related protein 4; MEF2C: myocyte enhancer factor 2C; MSCs: mesenchymal stem cells; MINPP1: multiple inositol polyphosphate phosphatase 1; MMP: matrix metalloprotease; MYBBP1A: MYB-binding protein 1a; PDLIM7: PDZ and LIM domain 7; PEI: polyethylenimine; PEX7: peroxisomal biogenesis factor 7; PLA: polylactide; RHOA: Ras homolog family member A; ROR2: RTK-like orphan receptor 2; RPL38: ribosomal protein L38; RUNX2: runt-related transcription factor 2; SAMD9: sterile alpha motif domain containing protein 9; MSCs: mesenchymal stem cells; SFRP1: secreted frizzled-related protein 1; SIX2: SIX homeobox 2; SMAD1: SMAD family member 1;SNAI1: snail family transcriptional repressor 1; SOX: sex-determining region Y-box; SUCO: SUN domain-containing ossification factor; TFIP11: tuftelin-interacting protein 11; TGF-*β*: transforming growth factor-*β*; TOB2: transducer of ERBB2; TUFT1: tuftelin 1; TWSG1: twisted gastrulation BMP signaling modulator 1; USP9X: USP9X protein variant; VEGF: vascular endothelial growth factor; VEGFR2: vascular endothelial growth factor receptor 2; WISP2: WNT1-inducible signaling pathway protein 2; ZNF423: zinc finger protein 423; ZNF521: ZNF521 protein.

## Supplementary Materials

Supplementary Materials Table S1: summary of included studies, citing secretome source, target disease/tissue, study model, and outcomes:

## References

[1] Atala A. (2008). Advances in tissue and organ replacement. *Current Stem Cell Research & Therapy*.

[2] Berthiaume F., Maguire T. J., Yarmush M. L. (2011). Tissue engineering and regenerative medicine: history, progress, and challenges. *Annual Review of Chemical and Biomolecular Engineering*.

[3] Stoltz J.-F., de Isla N., Li Y. P. (2015). Stem cells and regenerative medicine: myth or reality of the 21th century. *Stem Cells International*.

[4] Gao G., Cui X. (2016). Three-dimensional bioprinting in tissue engineering and regenerative medicine. *Biotechnology Letters*.

[5] Guan X., Avci‐Adali M., Alarçin E. (2017). Development of hydrogels for regenerative engineering. *Biotechnology Journal*.

[6] Rezwan K., Chen Q. Z., Blaker J. J., Boccaccini A. R. (2006). Biodegradable and bioactive porous polymer/inorganic composite scaffolds for bone tissue engineering. *Biomaterials*.

[7] Pina S., Oliveira J. M., Reis R. L. (2015). Natural-based nanocomposites for bone tissue engineering and regenerative medicine: a review. *Advanced Materials*.

[8] Qu H., Fu H., Han Z., Sun Y. (2019). Biomaterials for bone tissue engineering scaffolds: a review. *RSC Advances*.

[9] Gomes M. E., Reis R. L. (2004). Tissue engineering: key elements and some trends. *Macromolecular Bioscience*.

[10] Mallick S., Tripathi S., Srivastava P. (2015). Advancement in scaffolds for bone tissue engineering: a review. *IOSR Journal of Pharmacy and Biological Sciences Version*.

[11] Drahansky M., Paridah M., Moradbak A. (2016). Dentin materials as biological scaffolds for tissue engineering. *Intech*.

[12] Chocholata P., Kulda V., Babuska V. (2019). Fabrication of scaffolds for bone-tissue regeneration. *Materials*.

[13] Bose S., Roy M., Bandyopadhyay A. (2012). Recent advances in bone tissue engineering scaffolds. *Trends in Biotechnology*.

[14] Pilia M., Guda T., Appleford M. (2013). Development of composite scaffolds for load-bearing segmental bone defects. *BioMed Research International*.

[15] Roseti L., Parisi V., Petretta M. (2017). Scaffolds for bone tissue engineering: state of the art and new perspectives. *Materials Science and Engineering: C*.

[16] Kerativitayanan P., Tatullo M., Khariton M., Joshi P., Perniconi B., Gaharwar A. K. (2017). Nanoengineered osteoinductive and elastomeric scaffolds for bone tissue engineering. *ACS Biomaterials Science & Engineering*.

[17] He M., Callanan A. (2013). Comparison of methods for whole-organ decellularization in tissue engineering of bioartificial organs. *Tissue Engineering Part B: Reviews*.

[18] Scarritt M. E., Pashos N. C., Ba B. (2015). A review of cellularization strategies for tissue engineering of whole organs. *Frontiers in Bioengineering and Biotechnology*.

[19] Paduano F., Marrelli M., Alom N. (2017). Decellularized bone extracellular matrix and human dental pulp stem cells as a construct for bone regeneration. *Journal of Biomaterials Science, Polymer Edition*.

[20] Taylor D. A., Parikh R. B., Sampaio L. C. (2017). Bioengineering hearts: simple yet complex. *Current Stem Cell Reports*.

[21] Egusa H., Sonoyama W., Nishimura M., Atsuta I., Akiyama K. (2012). Stem cells in dentistry - Part I: Stem cell sources. *Journal of Prosthodontic Research*.

[22] Shah R., Hiremutt D., Jajoo S., Kamble A. (2016). Dental tissue engineering : future of regenerative dentistry. *Journal of Dental Research and Scientific Development*.

[23] Pires A. O., Mendes-Pinheiro B., Teixeira F. G. (2016). Unveiling the differences of secretome of human bone marrow mesenchymal stem cells, adipose tissue-derived stem cells, and human umbilical cord perivascular cells: a proteomic analysis. *Stem Cells and Development*.

[24] Hsiao S. T.-F., Asgari A., Lokmic Z. (2012). Comparative analysis of paracrine factor expression in human adult mesenchymal stem cells derived from bone marrow, adipose, and dermal tissue. *Stem Cells and Development*.

[25] Jones E. A., English A., Henshaw K. (2004). Enumeration and phenotypic characterization of synovial fluid multipotential mesenchymal progenitor cells in inflammatory and degenerative arthritis. *Arthritis and Rheumatism*.

[26] Caplan A. I. (2007). Adult mesenchymal stem cells for tissue engineering versus regenerative medicine. *Journal of Cellular Physiology*.

[27] Cicconetti A., Sacchetti B., Bartoli A. (2007). Human maxillary tuberosity and jaw periosteum as sources of osteoprogenitor cells for tissue engineering. *Oral Surgery, Oral Medicine, Oral Pathology, Oral Radiology, and Endodontology*.

[28] Bermudez M. A., Sendon-Lago J., Eiro N. (2015). Corneal epithelial wound healing and bactericidal effect of conditioned medium from human uterine cervical stem cells. *Investigative Ophthalmology & Visual Science*.

[29] Bermudez M. A., Sendon-Lago J., Seoane S. (2016). Anti-inflammatory effect of conditioned medium from human uterine cervical stem cells in uveitis. *Experimental Eye Research*.

[30] Osugi M., Katagiri W., Yoshimi R., Inukai T., Hibi H., Ueda M. (2012). Conditioned media from mesenchymal stem cells enhanced bone regeneration in rat calvarial bone defects. *Tissue engineering Part A*.

[31] Vizoso F. J., Eiro N., Cid S., Schneider J., Perez-Fernandez R. (2017). Mesenchymal stem cell secretome: toward cell-free therapeutic strategies in regenerative medicine. *International Journal of Molecular Sciences*.

[32] Fawzy El-Sayed K. M., Dorfer C., Fandrich F., Gieseler F., Moustafa M. H., Ungefroren H. (2012). Adult mesenchymal stem cells explored in the dental field. *Mesenchymal Stem Cells - Basics and Clinical Application II*.

[33] Fawzy El-Sayed K. M., Dorfer C., Fandrich F., Gieseler F., Moustafa M. H., Ungefroren H. (2013). Erratum to: adult mesenchymal stem cells explored in the dental field. *Mesenchymal Stem Cells - Basics and Clinical Application II*.

[34] Gronthos S., Mankani M., Brahim J., Robey P. G., Shi S. (2000). Postnatal human dental pulp stem cells (DPSCs) *in vitro* and *in vivo*. *Proceedings of the National Academy of Sciences*.

[35] Miura M., Gronthos S., Zhao M. (2003). SHED: stem cells from human exfoliated deciduous teeth. *Proceedings of the National Academy of Sciences*.

[36] Stanko P., Altanerova U., Jakubechova J., Repiska V., Altaner C. (2018). Dental mesenchymal stem/stromal cells and their exosomes. *Stem Cells International*.

[37] Seo B.-M., Miura M., Gronthos S. (2004). Investigation of multipotent postnatal stem cells from human periodontal ligament. *The Lancet*.

[38] Jo Y.-Y., Lee H.-J., Kook S.-Y. (2007). Isolation and characterization of postnatal stem cells from human dental tissues. *Tissue Engineering*.

[39] Morsczeck C., Götz W., Schierholz J. (2005). Isolation of precursor cells (PCs) from human dental follicle of wisdom teeth. *Matrix Biology*.

[40] Fawzy El-Sayed K. M., Paris S., Becker S. (2012). Isolation and characterization of multipotent postnatal stem/progenitor cells from human alveolar bone proper. *Journal of Cranio-Maxillofacial Surgery*.

[41] Fawzy El-Sayed K. M., Boeckler J., Dorfer C. E. (2017). TLR expression profile of human alveolar bone proper-derived stem/progenitor cells and osteoblasts. *Journal of Cranio-Maxillofacial Surgery*.

[42] Fawzy El-Sayed K. M., Dorfer C., Ungefroren H., Kassem N., Wiltfang J., Paris S. (2014). Effect of Emdogain enamel matrix derivative and BMP-2 on the gene expression and mineralized nodule formation of alveolar bone proper-derived stem/progenitor cells. *Journal of Cranio-Maxillofacial Surgery*.

[43] Sonoyama W., Liu Y., Fang D. (2006). Mesenchymal stem cell-mediated functional tooth regeneration in swine. *PloS One*.

[44] Ikeda E., Yagi K., Kojima M. (2008). Multipotent cells from the human third molar: feasibility of cell-based therapy for liver disease. *Differentiation*.

[45] Palmer R. M., Lubbock M. J. (1995). The soft connective tissues of the gingiva and periodontal ligament: are they unique?. *Oral Diseases*.

[46] Fawzy-El-Sayed K., Mekhemar M., Adam-Klages S., Kabelitz D., Dorfer C. (2016). TlR expression profile of human gingival margin-derived stem progenitor cells. *Medicina Oral Patología Oral y Cirugia Bucal*.

[47] Fawzy El-Sayed K. M., Hein D., Dorfer C. E. (2019). Retinol/inflammation affect stemness and differentiation potential of gingival stem/progenitor cells via Wnt/*β*-catenin. *Journal of Periodontal Research*.

[48] Fawzy El-Sayed K. M., Dorfer C. E. (2016). Gingival mesenchymal stem/progenitor cells: a unique tissue engineering gem. *Stem Cells International*.

[49] Fawzy El-Sayed K. M., Paris S., Graetz C. (2015). Isolation and characterisation of human gingival margin-derived STRO-1/MACS^+^ and MACS^−^ cell populations. *International Journal of Oral Science*.

[50] Alongi D. J., Yamaza T., Song Y. (2010). Stem/progenitor cells from inflamed human dental pulp retain tissue regeneration potential. *Regenerative Medicine*.

[51] Malekfar A., Valli K. S., Kanafi M. M., Bhonde R. R. (2016). Isolation and characterization of human dental pulp stem cells from cryopreserved pulp tissues obtained from teeth with irreversible pulpitis. *Journal of Endodontics*.

[52] Marrelli M., Paduano F., Tatullo M. (2013). Cells isolated from human periapical cysts express mesenchymal stem cell-like properties. *International Journal of Biological Sciences*.

[53] Tatullo M., Codispoti B., Pacifici A. (2017). Potential use of human periapical cyst-mesenchymal stem cells (hPCy-MSCs) as a novel stem cell source for regenerative medicine applications. *Frontiers in Cell and Developmental Biology*.

[54] Huang G. T. J., Gronthos S., Shi S. (2009). Mesenchymal stem cells derived from dental tissues vs. those from other sources: their biology and role in regenerative medicine. *Journal of Dental Research*.

[55] Cordeiro M. M., Dong Z., Kaneko T. (2008). Dental pulp tissue engineering with stem cells from exfoliated deciduous teeth. *Journal of Endodontics*.

[56] Dominici M., Le Blanc K., Mueller I. (2006). Minimal criteria for defining multipotent mesenchymal stromal cells. The International Society for Cellular Therapy position statement. *Cytotherapy*.

[57] Huang G. T. J., Yamaza T., Shea L. D. (2010). Stem/progenitor cell–mediated de novo regeneration of dental pulp with newly deposited continuous layer of dentin in an in vivo model. *Tissue engineering Part A*.

[58] Leucht P., Kim J.-B., Amasha R., James A. W., Girod S., Helms J. A. (2008). Embryonic origin and Hox status determine progenitor cell fate during adult bone regeneration. *Development*.

[59] Liu J., Yu F., Sun Y. (2015). Concise reviews: characteristics and potential applications of human dental tissue-derived mesenchymal stem cells. *Stem Cells*.

[60] Nakashima M., Iohara K. (2014). Mobilized dental pulp stem cells for pulp regeneration: initiation of clinical trial. *Journal of Endodontics*.

[61] Park C. H., Rios H. F., Jin Q. (2010). Biomimetic hybrid scaffolds for engineering human tooth-ligament interfaces. *Biomaterials*.

[62] Murakami M., Hayashi Y., Iohara K., Osako Y., Hirose Y., Nakashima M. (2015). Trophic effects and regenerative potential of mobilized mesenchymal stem cells from bone marrow and adipose tissue as alternative cell sources for pulp/dentin regeneration. *Cell Transplantation*.

[63] Kumar A., Kumar V., Rattan V., Jha V., Bhattacharyya S. (2018). Secretome proteins regulate comparative osteogenic and adipogenic potential in bone marrow and dental stem cells. *Biochimie*.

[64] Kumar A., Kumar V., Rattan V., Jha V., Pal A., Bhattacharyya S. (2017). Molecular spectrum of secretome regulates the relative hepatogenic potential of mesenchymal stem cells from bone marrow and dental tissue. *Scientific Reports*.

[65] Kumar A., Kumar V., Rattan V., Jha V., Bhattacharyya S. (2017). Secretome cues modulate the neurogenic potential of bone marrow and dental stem cells. *Molecular Neurobiology*.

[66] Mead B., Logan A., Berry M., Leadbeater W., Scheven B. A. (2014). Paracrine-mediated neuroprotection and neuritogenesis of axotomised retinal ganglion cells by human dental pulp stem cells: comparison with human bone marrow and adipose-derived mesenchymal stem cells. *PLoS One*.

[67] Davies O. G., Cooper P. R., Shelton R. M., Smith A. J., Scheven B. A. (2015). A comparison of the in vitro mineralisation and dentinogenic potential of mesenchymal stem cells derived from adipose tissue, bone marrow and dental pulp. *Journal of Bone and Mineral Metabolism*.

[68] Isobe Y., Koyama N., Nakao K. (2016). Comparison of human mesenchymal stem cells derived from bone marrow, synovial fluid, adult dental pulp, and exfoliated deciduous tooth pulp. *International Journal of Oral and Maxillofacial Surgery*.

[69] Song M., Lee J.-H., Bae J., Bu Y., Kim E.-C. (2017). Human dental pulp stem Cells are more effective than human bone marrow-derived mesenchymal stem cells in cerebral ischemic injury. *Cell Transplantation*.

[70] Abdullah M. F., Abdullah S. F., Omar N. S. (2014). Proliferation rate of stem cells derived from human dental pulp and identification of differentially expressed genes. *Cell Biology International*.

[71] Wang X., Sha X.-J., Li G.-H. (2012). Comparative characterization of stem cells from human exfoliated deciduous teeth and dental pulp stem cells. *Archives of Oral Biology*.

[72] Ankrum J., Karp J. M. (2010). Mesenchymal stem cell therapy: two steps forward, one step back. *Trends in Molecular Medicine*.

[73] Baglio S. R., Pegtel D. M., Baldini N. (2012). Mesenchymal stem cell secreted vesicles provide novel opportunities in (stem) cell-free therapy. *Frontiers in Physiology*.

[74] Maguire G. (2014). Stem cell therapy without the cells. *Communicative & Integrative Biology*.

[75] Wollert K. C., Drexler H. (2010). Cell therapy for the treatment of coronary heart disease: a critical appraisal. *Nature Reviews Cardiology*.

[76] Li M., Guo K., Ikehara S. (2014). Stem cell treatment for Alzheimer’s disease. *International Journal of Molecular Sciences*.

[77] Ranganath S. H., Levy O., Inamdar M. S., Karp J. M. (2012). Harnessing the mesenchymal stem cell secretome for the treatment of cardiovascular disease. *Cell Stem Cell*.

[78] Bai L., Li D., Li J. (2016). Bioactive molecules derived from umbilical cord mesenchymal stem cells. *Acta Histochemica*.

[79] Baraniak P. R., McDevitt T. C. (2010). Stem cell paracrine actions and tissue regeneration. *Regenerative Medicine*.

[80] Beer L., Mildner M., Ankersmit H. J. (2017). Cell secretome based drug substances in regenerative medicine: when regulatory affairs meet basic science. *Annals of Translational Medicine*.

[81] Katagiri W., Osugi M., Kawai T., Ueda M. (2013). Novel cell-free regeneration of bone using stem cell–derived growth factors. *The International Journal of Oral & Maxillofacial Implants*.

[82] Madrigal M., Rao K. S., Riordan N. H. (2014). A review of therapeutic effects of mesenchymal stem cell secretions and induction of secretory modification by different culture methods. *Journal of Translational Medicine*.

[83] Ciapetti G., Granchi D., Baldini N. (2012). The combined use of mesenchymal stromal cells and scaffolds for bone repair. *Current Pharmaceutical Design*.

[84] Phelps J., Sanati-Nezhad A., Ungrin M., Duncan N. A., Sen A. (2018). Bioprocessing of Mesenchymal Stem Cells and Their Derivatives: Toward Cell-Free Therapeutics. *Stem Cells International*.

[85] Sakai A., Ohshima M., Sugano N., Otsuka K., Ito K. (2006). Profiling the cytokines in gingival crevicular fluid using a cytokine antibody array. *Journal of Periodontology*.

[86] Assoni A., Coatti G., Valadares M. C. (2017). Different donors mesenchymal stromal cells secretomes reveal heterogeneous profile of relevance for therapeutic use. *Stem Cells and Development*.

[87] Pawitan J. A. (2014). Prospect of stem cell conditioned medium in regenerative medicine. *BioMed Research International*.

[88] Lee Y., El Andaloussi S., Wood M. J. A. (2012). Exosomes and microvesicles: extracellular vesicles for genetic information transfer and gene therapy. *Human Molecular Genetics*.

[89] Raposo G., Stoorvogel W. (2013). Extracellular vesicles: exosomes, microvesicles, and friends. *The Journal of Cell Biology*.

[90] Skog J., Würdinger T., van Rijn S. (2008). Glioblastoma microvesicles transport RNA and proteins that promote tumour growth and provide diagnostic biomarkers. *Nature Cell Biology*.

[91] Kim H. O., Choi S.-M., Kim H.-S. (2013). Mesenchymal stem cell-derived secretome and microvesicles as a cell-free therapeutics for neurodegenerative disorders. *Tissue Engineering and Regenerative Medicine*.

[92] Valadi H., Ekstrom K., Bossios A., Sjostrand M., Lee J. J., Lotvall J. O. (2007). Exosome-mediated transfer of mRNAs and microRNAs is a novel mechanism of genetic exchange between cells. *Nature Cell Biology*.

[93] Nakamura Y., Miyaki S., Ishitobi H. (2015). Mesenchymal-stem-cell-derived exosomes accelerate skeletal muscle regeneration. *FEBS Letters*.

[94] Tomasoni S., Longaretti L., Rota C. (2013). Transfer of growth factor receptor mRNA via exosomes unravels the regenerative effect of mesenchymal stem cells. *Stem Cells and Development*.

[95] Mathivanan S., Ji H., Simpson R. J. (2010). Exosomes: extracellular organelles important in intercellular communication. *Journal of Proteomics*.

[96] Ratajczak J., Miekus K., Kucia M. (2006). Embryonic stem cell-derived microvesicles reprogram hematopoietic progenitors: evidence for horizontal transfer of mRNA and protein delivery. *Leukemia*.

[97] György B., Szabó T. G., Pásztói M. (2011). Membrane vesicles, current state-of-the-art: emerging role of extracellular vesicles. *Cellular and Molecular Life Sciences*.

[98] Vishnubhatla I., Corteling R., Stevanato L., Hicks C., Sinden J. (2014). The development of stem cell-derived exosomes as a cell-free regenerative medicine. *Journal of Circulating Biomarkers*.

[99] Collino F., Deregibus M. C., Bruno S. (2010). Microvesicles derived from adult human bone marrow and tissue specific mesenchymal stem cells shuttle selected pattern of miRNAs. *PloS One*.

[100] Tkach M., Thery C. (2016). Communication by extracellular vesicles: where we are and where we need to go. *Cell*.

[101] Pant S., Hilton H., Burczynski M. E. (2012). The multifaceted exosome: biogenesis, role in normal and aberrant cellular function, and frontiers for pharmacological and biomarker opportunities. *Biochemical Pharmacology*.

[102] Biancone L., Bruno S., Deregibus M. C., Tetta C., Camussi G. (2012). Therapeutic potential of mesenchymal stem cell-derived microvesicles. *Nephrology Dialysis Transplantation*.

[103] Tachida Y., Sakurai H., Okutsu J. (2015). Proteomic comparison of the secreted factors of mesenchymal stem cells from bone marrow, adipose tissue and dental pulp. *Journal of Proteomics & Bioinformatics*.

[104] Yu S., Zhao Y., Ma Y., Ge L. (2016). Profiling the secretome of human stem cells from dental apical papilla. *Stem Cells and Development*.

[105] Joo K. H., Song J. S., Kim S. (2018). Cytokine expression of stem cells originating from the apical complex and coronal pulp of immature teeth. *Journal of Endodontics*.

[106] Ishizaka R., Hayashi Y., Iohara K. (2013). Stimulation of angiogenesis, neurogenesis and regeneration by side population cells from dental pulp. *Biomaterials*.

[107] Hayashi Y., Murakami M., Kawamura R., Ishizaka R., Fukuta O., Nakashima M. (2015). CXCL14 and MCP1 are potent trophic factors associated with cell migration and angiogenesis leading to higher regenerative potential of dental pulp side population cells. *Stem Cell Research & Therapy*.

[108] Nakamura S., Yamada Y., Katagiri W., Sugito T., Ito K., Ueda M. (2009). Stem cell proliferation pathways comparison between human exfoliated deciduous teeth and dental pulp stem cells by gene expression profile from promising dental pulp. *Journal of Endodontics*.

[109] Sakai K., Yamamoto A., Matsubara K. (2012). Human dental pulp-derived stem cells promote locomotor recovery after complete transection of the rat spinal cord by multiple neuro-regenerative mechanisms. *The Journal of Clinical Investigation*.

[110] Sugimura-Wakayama Y., Katagiri W., Osugi M. (2015). Peripheral nerve regeneration by secretomes of stem cells from human exfoliated deciduous teeth. *Stem Cells and Development*.

[111] Kano F., Matsubara K., Ueda M., Hibi H., Yamamoto A. (2017). Secreted ectodomain of sialic acid-binding Ig-like lectin-9 and monocyte chemoattractant protein-1 synergistically regenerate transected rat peripheral nerves by altering macrophage polarity. *Stem Cells*.

[112] Matsubara K., Matsushita Y., Sakai K. (2015). Secreted ectodomain of sialic acid-binding Ig-like lectin-9 and monocyte chemoattractant protein-1 promote recovery after rat spinal cord injury by altering macrophage polarity. *Journal of Neuroscience*.

[113] Yamagata M., Yamamoto A., Kako E. (2013). Human dental pulp-derived stem cells protect against hypoxic-ischemic brain injury in neonatal mice. *Stroke*.

[114] Inoue T., Sugiyama M., Hattori H., Wakita H., Wakabayashi T., Ueda M. (2013). Stem cells from human exfoliated deciduous tooth-derived conditioned medium enhance recovery of focal cerebral ischemia in rats. *Tissue Engineering Part A*.

[115] Li Y., Yang Y.-Y., Ren J.-L., Xu F., Chen F.-M., Li A. (2017). Exosomes secreted by stem cells from human exfoliated deciduous teeth contribute to functional recovery after traumatic brain injury by shifting microglia M1/M2 polarization in rats. *Stem Cell Research & Therapy*.

[116] Asadi-Golshan R., Razban V., Mirzaei E. (2018). Sensory and motor behavior evidences supporting the usefulness of conditioned medium from dental pulp-derived stem cells in spinal cord injury in rats. *Asian Spine Journal*.

[117] Mita T., Furukawa-Hibi Y., Takeuchi H. (2015). Conditioned medium from the stem cells of human dental pulp improves cognitive function in a mouse model of Alzheimer's disease. *Behavioural Brain Research*.

[118] Shimojima C., Takeuchi H., Jin S. (2016). Conditioned medium from the stem cells of human exfoliated deciduous teeth ameliorates experimental autoimmune encephalomyelitis. *Journal of Immunology*.

[119] Fujii H., Matsubara K., Sakai K. (2015). Dopaminergic differentiation of stem cells from human deciduous teeth and their therapeutic benefits for Parkinsonian rats. *Brain Research*.

[120] Jarmalaviciute A., Tunaitis V., Pivoraite U., Venalis A., Pivoriunas A. (2015). Exosomes from dental pulp stem cells rescue human dopaminergic neurons from 6-hydroxy-dopamine-induced apoptosis. *Cytotherapy*.

[121] Narbute K., Piļipenko V., Pupure J. (2019). Intranasal administration of extracellular vesicles derived from human teeth stem cells improves motor symptoms and normalizes tyrosine hydroxylase expression in the substantia nigra and striatum of the 6-hydroxydopamine-treated rats. *Stem Cells Translational Medicine*.

[122] Tsuruta T., Sakai K., Watanabe J., Katagiri W., Hibi H. (2018). Dental pulp-derived stem cell conditioned medium to regenerate peripheral nerves in a novel animal model of dysphagia. *PLoS One*.

[123] de Cara S. P. H. M., Origassa C. S. T., de Sá Silva F. (2019). Angiogenic properties of dental pulp stem cells conditioned medium on endothelial cells *in vitro* and in rodent orthotopic dental pulp regeneration. *Heliyon*.

[124] Hirata M., Ishigami M., Matsushita Y. (2016). Multifaceted therapeutic benefits of factors derived from dental pulp stem cells for mouse Liver Fibrosis. *Stem Cells Translational Medicine*.

[125] Matsushita Y., Ishigami M., Matsubara K. (2017). Multifaceted therapeutic benefits of factors derived from stem cells from human exfoliated deciduous teeth for acute liver failure in rats. *Journal of Tissue Engineering and Regenerative Medicine*.

[126] Wakayama H., Hashimoto N., Matsushita Y. (2015). Factors secreted from dental pulp stem cells show multifaceted benefits for treating acute lung injury in mice. *Cytotherapy*.

[127] Yamaguchi S., Shibata R., Yamamoto N. (2015). Dental pulp-derived stem cell conditioned medium reduces cardiac injury following ischemia-reperfusion. *Scientific Reports*.

[128] Izumoto-Akita T., Tsunekawa S., Yamamoto A. (2015). Secreted factors from dental pulp stem cells improve glucose intolerance in streptozotocin-induced diabetic mice by increasing pancreatic *β*-cell function. *BMJ Open Diabetes Research & Care*.

[129] Ishikawa J., Takahashi N., Matsumoto T. (2016). Factors secreted from dental pulp stem cells show multifaceted benefits for treating experimental rheumatoid arthritis. *Bone*.

[130] Luo P., Jiang C., Ji P., Wang M., Xu J. (2019). Exosomes of stem cells from human exfoliated deciduous teeth as an anti-inflammatory agent in temporomandibular joint chondrocytes via miR-100-5p/mTOR. *Stem Cell Research & Therapy*.

[131] Gunawardena T. N. A., Masoudian Z., Rahman M. T., Ramasamy T. S., Ramanathan A., Abu Kasim N. H. (2019). Dental derived stem cell conditioned media for hair growth stimulation. *PLoS One*.

[132] Yamada Y., Nakamura-Yamada S., Kusano K., Baba S. (2019). Clinical potential and current progress of dental pulp stem cells for various systemic diseases in regenerative medicine: a concise review. *International Journal of Molecular Sciences*.

[133] Geng Y. W., Zhang Z., Liu M. Y., Hu W. P. (2017). Differentiation of human dental pulp stem cells into neuronal by resveratrol. *Cell Biology International*.

[134] Yan X., Qin H., Qu C., Tuan R. S., Shi S., Huang G. T. J. (2010). iPS cells reprogrammed from human mesenchymal-like stem/progenitor cells of dental tissue origin. *Stem Cells and Development*.

[135] Tran-Hung L., Laurent P., Camps J., About I. (2008). Quantification of angiogenic growth factors released by human dental cells after injury. *Archives of Oral Biology*.

[136] Hilkens P., Fanton Y., Martens W. (2014). Pro-angiogenic impact of dental stem cells *in vitro* and *in vivo*. *Stem Cell Research*.

[137] Ratajczak J., Bronckaers A., Dillen Y. (2016). The neurovascular properties of dental stem cells and their importance in dental tissue engineering. *Stem Cells International*.

[138] Bronckaers A., Hilkens P., Fanton Y. (2013). Angiogenic properties of human dental pulp stem cells. *PloS One*.

[139] Bianco J., De Berdt P., Deumens R., des Rieux A. (2016). Taking a bite out of spinal cord injury: do dental stem cells have the teeth for it?. *Cellular and Molecular Life Sciences*.

[140] Rajput I. R., Hussain A., Li Y. L. (2014). Saccharomyces boulardii and bacillus subtilis B10 modulate TLRs mediated signaling to induce immunity by chicken BMDCs. *Journal of Cellular Biochemistry*.

[141] Kanafi M., Majumdar D., Bhonde R., Gupta P., Datta I. (2014). Midbrain cues dictate differentiation of human dental pulp stem cells towards functional dopaminergic neurons. *Journal of Cellular Physiology*.

[142] Chang C. C., Chang K. C., Tsai S. J., Chang H. H., Lin C. P. (2014). Neurogenic differentiation of dental pulp stem cells to neuron-like cells in dopaminergic and motor neuronal inductive media. *Journal of the Formosan Medical Association*.

[143] Zhang J., Lian M., Cao P. (2017). Effects of nerve growth factor and basic fibroblast growth factor promote human dental pulp stem cells to neural differentiation. *Neurochemical Research*.

[144] Yazid F., Luchman N. A., Wahab R. M. A., Ariffin S. H. Z. (2018). Comparison of Characterization and Osteoblast Formation Between Human Dental Pulp Stem Cells (hDPSC) and Stem Cells from Deciduous Teeth (SHED). *Proceedings of the Second International Conference on the Future of ASEAN (ICoFA) 2017 – Volume 2*.

[145] Hakki S. S., Kayis S. A., Hakki E. E. (2015). Comparison of mesenchymal stem cells isolated from pulp and periodontal ligament. *Journal of Periodontology*.

[146] Gervois P., Wolfs E., Dillen Y. (2017). Paracrine maturation and migration of SH-SY5Y cells by dental pulp stem cells. *Journal of Dental Research*.

[147] Yamamoto T., Osako Y., Ito M. (2016). Trophic effects of dental pulp stem cells on schwann cells in peripheral nerve regeneration. *Cell Transplantation*.

[148] Ahmed N. E.-M. B., Murakami M., Hirose Y., Nakashima M. (2016). Therapeutic potential of dental pulp stem cell secretome for Alzheimer’s disease treatment: an in vitro study. *Stem Cells International*.

[149] Wang J., Zuzzio K., Walker C. L. (2019). Systemic dental pulp stem cell secretome therapy in a mouse model of amyotrophic lateral sclerosis. *Brain Sciences*.

[150] Makino E., Nakamura N., Miyabe M. (2019). Conditioned media from dental pulp stem cells improved diabetic polyneuropathy through anti-inflammatory, neuroprotective and angiogenic actions: cell-free regenerative medicine for diabetic polyneuropathy. *Journal of Diabetes Investigation*.

[151] Chen T.-F., Chen K.-W., Chien Y. (2019). Dental pulp stem cell-derived factors alleviate subarachnoid hemorrhage-induced neuroinflammation and ischemic neurological deficits. *International Journal of Molecular Sciences*.

[152] Ma D., Ma Z., Zhang X. (2009). Effect of Age and Extrinsic Microenvironment on the Proliferation and Osteogenic Differentiation of Rat Dental Pulp Stem Cells *In Vitro*. *Journal of Endodontics*.

[153] Paschalidis T., Bakopoulou A., Papa P., Leyhausen G., Geurtsen W., Koidis P. (2014). Dental pulp stem cells' secretome enhances pulp repair processes and compensates TEGDMA-induced cytotoxicity. *Dental Materials*.

[154] Fujio M., Xing Z., Sharabi N. (2017). Conditioned media from hypoxic-cultured human dental pulp cells promotes bone healing during distraction osteogenesis. *Journal of Tissue Engineering and Regenerative Medicine*.

[155] Huang C.-C., Narayanan R., Alapati S., Ravindran S. (2016). Exosomes as biomimetic tools for stem cell differentiation: applications in dental pulp tissue regeneration. *Biomaterials*.

[156] Kawamura R., Hayashi Y., Murakami H., Nakashima M. (2016). EDTA soluble chemical components and the conditioned medium from mobilized dental pulp stem cells contain an inductive microenvironment, promoting cell proliferation, migration, and odontoblastic differentiation. *Stem Cell Research & Therapy*.

[157] Nakayama H., Iohara K., Hayashi Y., Okuwa Y., Kurita K., Nakashima M. (2017). Enhanced regeneration potential of mobilized dental pulp stem cells from immature teeth. *Oral Diseases*.

[158] Iohara K., Zheng L., Wake H. (2008). A novel stem cell source for vasculogenesis in ischemia: subfraction of side population cells from dental pulp. *Stem Cells*.

[159] Jin S. H., Lee J. E., Yun J. H., Kim I., Ko Y., Park J. B. (2015). Isolation and characterization of human mesenchymal stem cells from gingival connective tissue. *Journal of Periodontal Research*.

[160] Zhang Q. Z., Nguyen A. L., Yu W. H., Le A. D. (2012). Human oral mucosa and Gingiva. *Journal of Dental Research*.

[161] Fawzy El-Sayed K. M., Mekhemar M. K., Beck-Broichsitter B. E. (2015). Periodontal regeneration employing gingival margin-derived stem/progenitor cells in conjunction with IL-1ra-hydrogel synthetic extracellular matrix. *Journal of Clinical Periodontology*.

[162] Fawzy El-Sayed K. M., Paris S., Becker S. T. (2012). Periodontal regeneration employing gingival margin-derived stem/progenitor cells: an animal study. *Journal of Clinical Periodontology*.

[163] Fawzy El‐Sayed K. M., Elahmady M., Adawi Z. (2019). The periodontal stem/progenitor cell inflammatory-regenerative cross talk: a new perspective. *Journal of Periodontal Research*.

[164] Fawzy El-Sayed K., Graetz C., Kohnlein T., Mekhemar M., Dorfer C. (2018). Effect of total sonicated Aggregatibacter actinomycetemcomitans fragments on gingival stem/progenitor cells. *Medicina Oral Patología Oral y Cirugia Bucal*.

[165] Zhang F., Si M., Wang H., Mekhemar M. K., Dorfer C. E., Fawzy El-Sayed K. M. (2017). IL-1/TNF-*α* inflammatory and anti-inflammatory synchronization affects gingival stem/progenitor cells’ regenerative attributes. *Stem Cells International*.

[166] Mekhemar M. K., Adam-Klages S., Kabelitz D., Dorfer C. E., Fawzy El-Sayed K. M. (2018). TLR-induced immunomodulatory cytokine expression by human gingival stem/progenitor cells. *Cellular Immunology*.

[167] Xu X., Chen C., Akiyama K. (2013). Gingivae contain neural-crest- and mesoderm-derived mesenchymal stem cells. *Journal of Dental Research*.

[168] Mao Q., Nguyen P. D., Shanti R. M. (2019). Gingiva-derived mesenchymal stem cell-extracellular vesicles activate Schwann cell repair phenotype and promote nerve regeneration. *Tissue Engineering Part A*.

[169] Rao F., Zhang D., Fang T. (2019). Exosomes from human gingiva-derived mesenchymal stem cells combined with biodegradable chitin conduits promote rat sciatic nerve regeneration. *Stem Cells International*.

[170] Rajan T. S., Diomede F., Bramanti P., Trubiani O., Mazzon E. (2017). Conditioned medium from human gingival mesenchymal stem cells protects motor-neuron-like NSC-34 cells against scratch-injury-induced cell death. *International Journal of Immunopathology and Pharmacology*.

[171] Shi Q., Qian Z., Liu D. (2017). GMSC-derived exosomes combined with a chitosan/silk hydrogel sponge accelerates wound healing in a diabetic rat skin defect model. *Frontiers in Physiology*.

[172] Diomede F., Gugliandolo A., Scionti D. (2018). Biotherapeutic effect of gingival stem cells conditioned medium in bone tissue restoration. *International Journal of Molecular Sciences*.

[173] Zhang Y., Shi S., Xu Q., Zhang Q., Shanti R. M., Le A. D. (2019). SIS-ECM laden with GMSC-derived exosomes promote taste bud regeneration. *Journal of Dental Research*.

[174] Kou X., Xu X., Chen C. (2018). The Fas/Fap-1/Cav-1 complex regulates IL-1RA secretion in mesenchymal stem cells to accelerate wound healing. *Science Translational Medicine*.

[175] Rajan T. S., Giacoppo S., Diomede F. (2016). The secretome of periodontal ligament stem cells from MS patients protects against EAE. *Scientific Reports*.

[176] Giacoppo S., Thangavelu S. R., Diomede F. (2017). Anti-inflammatory effects of hypoxia-preconditioned human periodontal ligament cell secretome in an experimental model of multiple sclerosis: a key role of IL-37. *The FASEB Journal*.

[177] Diomede F., Gugliandolo A., Cardelli P. (2018). Three-dimensional printed PLA scaffold and human gingival stem cell-derived extracellular vesicles: a new tool for bone defect repair. *Stem Cell Research & Therapy*.

[178] McCulloch C. A. G., Bordin S. (1991). Role of fibroblast subpopulations in periodontal physiology and pathology. *Journal of Periodontal Research*.

[179] Isaka J., Ohazama A., Kobayashi M. (2001). Participation of periodontal ligament cells with regeneration of alveolar bone. *Journal of Periodontology*.

[180] Fawzy El-Sayed K. M., Dorfer C. E. (2017). Animal models for periodontal tissue engineering: a knowledge-generating process. *Tissue Engineering Part C: Methods*.

[181] Nagata M., Iwasaki K., Akazawa K. (2017). Conditioned medium from periodontal ligament stem cells enhances periodontal regeneration. *Tissue Engineering Part A*.

[182] Rajan T. S., Scionti D., Diomede F. (2017). Gingival stromal cells as an in vitro model: cannabidiol modulates genes linked with amyotrophic lateral sclerosis. *Journal of Cellular Biochemistry*.

[183] Sedgley C. M., Botero T. M. (2012). Dental stem cells and their sources. *Dental clinics of North America*.

[184] Achilleos A., Trainor P. A. (2012). Neural crest stem cells: discovery, properties and potential for therapy. *Cell Research*.

[185] Eleuterio E., Trubiani O., Sulpizio M. (2013). Proteome of human stem cells from periodontal ligament and dental pulp. *PloS One*.

[186] Menicanin D., Mrozik K. M., Wada N. (2014). Periodontal-ligament-derived stem cells exhibit the capacity for long-term survival, self-renewal, and regeneration of multiple tissue types in vivo. *Stem Cells and Development*.

[187] Tsumanuma Y., Iwata T., Washio K. (2011). Comparison of different tissue-derived stem cell sheets for periodontal regeneration in a canine 1-wall defect model. *Biomaterials*.

[188] Park J. C., Lee S. M., Kim J. C. (2012). Effect of humoral factors from hPDLSCs on the biologic activity of hABCs. *Oral Diseases*.

[189] Mizuno N., Ozeki Y., Shiba H. (2008). Humoral factors released from human periodontal ligament cells influence calcification and proliferation in human bone marrow mesenchymal stem cells. *Journal of Periodontology*.

[190] Kim K., Jeon M., Lee H. S. (2016). Comparative analysis of secretory factors from permanent- and deciduous-teeth periodontal ligament cells. *Archives of Oral Biology*.

[191] Ohshima M., Yamaguchi Y., Micke P., Abiko Y., Otsuka K. (2008). In vitro characterization of the cytokine profile of the epithelial cell rests of Malassez. *Journal of Periodontology*.

[192] Rajan T. S., Giacoppo S., Diomede F., Bramanti P., Trubiani O., Mazzon E. (2017). Human periodontal ligament stem cells secretome from multiple sclerosis patients suppresses NALP3 inflammasome activation in experimental autoimmune encephalomyelitis. *International Journal of Immunopathology and Pharmacology*.

[193] Pizzicannella J., Gugliandolo A., Orsini T. (2019). Engineered extracellular vesicles from human periodontal-ligament stem cells increase VEGF/VEGFR2 expression during bone regeneration. *Frontiers in Physiology*.

[194] Diomede F., D'Aurora M., Gugliandolo A. (2018). A novel role in skeletal segment regeneration of extracellular vesicles released from periodontal-ligament stem cells. *International Journal of Nanomedicine*.

[195] Kémoun P., Laurencin-Dalicieux S., Rue J. (2007). Human dental follicle cells acquire cementoblast features under stimulation by BMP-2/-7 and enamel matrix derivatives (EMD) in vitro. *Cell and Tissue Research*.

[196] Morsczeck C., Schmalz G., Reichert T. E., Vollner F., Galler K., Driemel O. (2008). Somatic stem cells for regenerative dentistry. *Clinical Oral Investigations*.

[197] Abe S., Yamaguchi S., Amagasa T. (2007). Multilineage cells from apical pulp of human tooth with immature apex. *Oral Science International*.

[198] Huang G. T. J., Sonoyama W., Liu Y., Liu H., Wang S., Shi S. (2008). The hidden treasure in apical papilla: the potential role in pulp/dentin regeneration and bioroot engineering. *Journal of Endodontics*.

[199] Hensley K., Robinson K. A., Gabbita S. P., Salsman S., Floyd R. A. (2000). Reactive oxygen species, cell signaling, and cell injury. *Free Radical Biology & Medicine*.

[200] Sies H., Berndt C., Jones D. P. (2017). Oxidative stress. *Annual Review of Biochemistry*.

[201] Liu C., Mo L., Niu Y., Li X., Zhou X., Xu X. (2017). The role of reactive oxygen species and autophagy in periodontitis and their potential linkage. *Frontiers in Physiology*.

[202] Di Meo S., Reed T. T., Venditti P., Victor V. M. (2016). Role of ROS and RNS sources in physiological and pathological conditions. *Oxidative Medicine and Cellular Longevity*.

[203] Tatullo M., Marrelli M., Scacco S. (2012). Relationship between oxidative stress and “burning mouth syndrome” in female patients: a scientific hypothesis. *European Review for Medical and Pharmacological Sciences*.

[204] Baum C., Modlich U., Göhring G., Schlegelberger B. (2011). Concise review: managing genotoxicity in the therapeutic modification of stem cells. *Stem Cells*.

[205] Duailibi M. T., Kulikowski L. D., Duailibi S. E. (2012). Cytogenetic instability of dental pulp stem cell lines. *Journal of Molecular Histology*.

[206] Batel R., Jakšić Ž., Bihari N. (1999). A Microplate Assay for DNA Damage Determination (*Fast Micromethod*)in Cell Suspensions and Solid Tissues. *Analytical Biochemistry*.

[207] Kim W.-S., Park B.-S., Kim H.-K. (2008). Evidence supporting antioxidant action of adipose-derived stem cells: protection of human dermal fibroblasts from oxidative stress. *Journal of Dermatological Science*.

[208] Arslan F., Lai R. C., Smeets M. B. (2013). Mesenchymal stem cell-derived exosomes increase ATP levels, decrease oxidative stress and activate PI3K/Akt pathway to enhance myocardial viability and prevent adverse remodeling after myocardial ischemia/reperfusion injury. *Stem Cell Research*.

[209] Song M., Jue S.-S., Cho Y.-A., Kim E.-C. (2015). Comparison of the effects of human dental pulp stem cells and human bone marrow-derived mesenchymal stem cells on ischemic human astrocytes in vitro. *Journal of Neuroscience Research*.

[210] Modo M., Rezaie P., Heuschling P., Patel S., Male D. K., Hodges H. (2002). Transplantation of neural stem cells in a rat model of stroke: assessment of short-term graft survival and acute host immunological response. *Brain Research*.

[211] Rubio D., Garcia S., Paz M. F. (2008). Molecular characterization of spontaneous mesenchymal stem cell transformation. *PloS One*.

