# Supplementary material for: Dental Stem Cell-Derived Secretome/Conditioned Medium: The Future for Regenerative Therapeutic Applications
Source: Stem Cells Int. 2020 Jan 31;2020:7593402. doi: 10.1155/2020/7593402 (PMC7013327; doi:10.1155/2020/7593402)
Supplement: Supplementary Materials — Table S1: summary of included studies, citing secretome source, target disease/tissue, study model, and outcomes: [file 7593402.f1.pdf]

Table S1: Summary of included studies, citing secretome source, target disease/tissue, study model and outcomes:

Authors/ year	Secretome source	Target disease/ tissue	Study model	Outcome
		**SHED-CM**		
**Sakai et al, 2012 [71]**	SHED-CM DPSCs-CM	Spinal cord injury	In vivo model of spinal cord injury. In vitro	Neurite extension was promoted through the inhibition of multiple axon growth inhibitors (AGI). Therefore, SHED-CM and DPSCs-CM can be beneficial in neural regeneration.
**Inoue et al., 2013 [69]**	SHED-CM	Cerebral stroke	In vivo stroke model in rats subjected to permanent middle cerebral artery occlusion (pMCAO).	Intranasal administration of SHED-CM promoted the migration and differentiation of endogenous neuronal progenitor cells, induced vasculogenesis and improved ischemic brain injury after pMCAO.
**Yamagata et al, 2013 [68]**	SHED-CM	Hypoxia-ischemia brain injury	In vivo hypoxic-ischemic brain injury mouse model.	Intracerebral administration of SHED-CM showed improved recovery of neurological function, mice survival rate and neuropathological score, providing neuroprotective microenvironment.
**Fujii et al, 2015 [75]**	SHED-CM	Neurons/Parkinson's disease	In vivo Parkinson's disease rat model. In vitro	SHED-CM showed potential therapeutic benefits for treating Parkinson's disease. It showed a neuroprotective effect that promoted neurite outgrowth of neurons and inhibited neuron apoptosis.
**Izumoto-Akita et al, 2015 [83]**	SHED-CM	Diabetes/Pancreatic β-cell	In vivo streptozotocin-induced diabetic mouse model.<br><br>In vitro culture of mouse pancreatic β-cell line (MIN6).	Intravenous administration of SHED-CM improved glucose intolerance, increased pancreatic insulin content and β-cell mass.<br><br>In vitro, SHED-CM enhanced insulin secretion in

				MIN6 cell culture and reduced STZ-induced cell death. SHED-CM presents a promising therapeutic approach in the treatment of diabetes.
**Jarmalaviciute et al., 2015 [76]**	SHEDs- EXs and MVs	Parkinson's disease/dopaminergic neurons	In vitro human dopaminergic neurons cell culture treated with 6-OHDA	SHED-EXs but not microvesicles have grown on the laminin-coated 3D alginate micro-carriers suppressed 6-OHDA-induced apoptosis in dopaminergic neurons. SHED-EXs was shown to have potent neuroprotective properties and could be a potential therapeutic agent in the treatment of Parkinson's disease.
**Matsubara et al., 2015 [67]**	SHED-CM	Spinal cord injury	In vivo rat injured spinal cord	Intrathecal administration of SHED-CM into rat injured spinal cord during the acute postinjury period caused remarkable recovery of hindlimb locomotor function. Due to the combined effect of monocyte chemoattractant protein-1 (MCP-1) and the secreted ectodomain of sialic acid-binding Ig-like lectin-9 (ED-Siglec-9), from SHED-CM, that induced anti-inflammatory M2-like macrophage.
**Mita et al, 2015 [73]**	SHED-CM	Alzheimer's disease (AD)	In vivo mouse model of Alzheimer's disease.	Intranasal administration SHED-CM attenuated the pro-inflammatory responses induced by β-amyloid plaques and generated an anti-inflammatory/neuro-reparative environment. SHED-CM is rich in multiple neuroregenerative factors required for the treatment of cognitive deficits, by improving neurotransmission, neuroprotection, axonal elongation, M2-like microglial activation and suppression of inflammation.
**Sugimura-Wakayama et al., 2015 [65]**	SHED-CM	Peripheral (Sciatic) nerve regeneration	In vivo rats' sciatic nerve defect model.	SHED-CM promoted sciatic nerve regeneration, axon regeneration, remyelination, motor functional recovery and prevented muscle

				atrophy in the rat model.
			In vitro Schwann cells culture.	SHED-CM increased axon growth, peripheral nerve tissue angiogenesis, Schwann cells proliferation, migration as well as activation and neuronal cell survival.
**Wakayama et al., 2015 [81]**	SHED-CM	Lung acute respiratory distress syndrome (ARDS)	In vivo bleomycin (BLM)-induced acute lung injury mouse model.	A single IV infusion of SHED-CM diminished the BLM-induced pro-inflammatory response and produced an anti-inflammatory/tissue-regenerative environment by induction of anti-inflammatory M2-like lung macrophages. SHED-CM showed therapeutic potential for treating bleomycin-induced mice acute lung injury.
**Yamaguchi et al, 2015 [82]**	SHED-CM	Acute myocardial infarction	In vivo mouse model of ischemia-reperfusion (I/R).	Intravenous injection of SHED-CM reduced the size of myocardial infarct, myocyte apoptosis and inflammatory cytokine levels, such as TNF-α, IL-6 and IL-β, in the myocardium following I/R.
			In vitro Cardiac myocytes culture.	SHED-CM significantly reduced LPS-induced expression of pro-inflammatory genes and suppressed apoptosis under hypoxia/serum-deprivation.
**Hirata et al., 2016 [79]**	SHED-CM	Liver fibrosis	In vivo mouse carbon tetrachloride (ccl4)-induced liver fibrosis model.	A single intravenous dose of SHED-CM suppressed chronic inflammation, protected against hepatocytes apoptosis while eliminating activated hepatic stellate cells and induced differentiation of tissue-repairing macrophages.
**Ishikawa et al., 2016 [84]**	SHED-CM	Rheumatoid arthritis (RA)	In vivo anti-collagen type II antibody-induced arthritis (CAIA), a mouse model of RA.	Single IV administration of SHED-CM provided therapeutic factors for treating CAIA including ED-Siglec-9-dependent induction of M2 macrophage polarization, inhibition of osteoclastogenesis and bone destruction.

**Shimojima et al., 2016 [74]**	SHED-CM	Multiple sclerosis (MS)	In vivo multiple sclerosis mouse model.	A single injection of SHED-CM or ED–Siglec-9 significantly improved experimental autoimmune encephalomyelitis (EAE) by improving disease scores, reduced demyelination and axonal injury, reduced inflammatory cell infiltration and pro-inflammatory cytokine expression in the spinal cord, through shifting in the microglia/macrophage phenotype from M1 to M2.
**Kano et al, 2017 [66]**	SHED-CM	Peripheral nerves	In vitro rat model of facial nerve transection.	MCP-1/sSiglec-9 in SHED-CM mediated neurological regeneration through inducing polarization of tissue-repairing macrophages, Schwann-cell bridging instead of scar formation, axonal regeneration and restoration of nerve function.<br>Those finding suggests the potential role of SHED-CM in the regenerative treatment of severely injured peripheral nerves.
**Li et al., 2017 [70]**	SHED- EXs	Traumatic brain injury (TBI)	In vivo rat traumatic brain injury (TBI) model<br><br>In vitro co-culture activated BV-2 microglia cells with SHED-EXs.	Local injection of SHED-EXs improved rat motor functional recovery and reduced cortical lesion. SHED-EXs reduced neuroinflammation by shifting microglia M1/M2 polarization.
**Matsushita et al., 2017 [80]**	SHED-CM	Acute liver failure (ALF)	In vivo d-galactosamine-induced rat model of acute liver failure (ALF)	A single IV administration SHED-CM noticeably enhanced the condition of the injured liver and the animals' survival rate. SHED-CM generated an anti-inflammatory/tissue-regenerating environment, together with the induction of anti-inflammatory M2-like hepatic macrophages.
**Asadi-Golshan et al, 2018 [72]**	SHED-CM	Spinal cord injury (SCI)	In vivo rat spinal cord injury model.	Intraspinal administration of collagen hydrogel containing SHED-CM led to enhanced

				neurological functional recovery.
**Tsuruta et al., 2018 [78]**	SHED-CM	Superior laryngeal nerve (SLN) injury	In vivo superior laryngeal nerve injury dysphagia rat model.	Injection of SHED-CM in the rat tail vein improved the functional recovery of the SLN and significantly promoted axonal regeneration via converting pro-inflammatory M1 macrophages to the anti-inflammatory M2 phenotype and enhanced angiogenesis at the injury site.
**de Cara et al., 2019 [87]**	SHED-CM	Dental pulp regeneration	In vivo rodent orthotopic model of dental pulp regeneration in rats.<br><br>In vitro on human umbilical vein endothelial cells (HUVEC) culture.	SHED-CM showed the formation of connective tissue similar to the dental pulp inside the root canal and promoted angiogenesis.<br><br>SHED-CM promoted angiogenesis, reduced apoptosis while increased migration and vascular-like structures formation due to the presence of VEGF in SHED-CM.
**Gunawardena et al., 2019 [86]**	SHED-CM	Alopecia	In vivo telogen-staged-synchronized C3H/HeN female mouse model.<br><br>In vitro SHED and hair follicle stem cells (HFSCs) culture.	Three sub-cutaneous injections of SHED-CM resulted in significantly faster stimulation of hair growth.<br><br>SHED-CM resulted in a significantly higher number of anagen-staged hair follicles and a significantly lower number of telogen-staged hair follicles.
**Luo et al, 2019 [85]**	SHED- EXs	Temporomandibular joint osteoarthritis	In vitro TMJ osteoarthritis model induced in human chondrocytes culture.	SHED-EXs suppressed the expression of IL-6, IL-8, MMP1, MMP3, MMP9, MMP13 and ADAMTS5. miR-100, being an abundant miRNA in SHED-EXs, suppressed inflammation via repression of mTOR. Thus, SHED-EXs could suppress inflammation in TMJ osteoarthritis.
**Narbute et al, 2019 [77]**	SHED-EVs	Parkinson's disease (PD)	In vivo unilateral 6-hydroxydopamine (6-OHDA) medial forebrain bundle (MFB) rat model of	Intranasal administration of SHED-EVs showed effective suppression of gait impairments and normalization of tyrosine hydroxylase expression in striatum and substantia nigra of rats.

			Parkinson's disease.	
**DPSCS-CM**				
**Iohara et al., 2008 [108]**	DPSCs-CM	Angiogenesis	In vitro culture of HUVECs	DPSCs-CM provided mitogenic and anti-apoptotic activities on HUVECs similar to the effect of MMP3, VEGF-A and G-CSF.
**Bronckaers et al., 2013 [107]**	DPSCs-CM	Angiogenesis	In vitro culture of human dental pulp cells.	Antibody array showed the existence of a wide array of pro-and anti-angiogenic factors; including VEGF, MCP-1, PAI-1 and endostatin in DPSC-CM.
			In vitro culture of human microvascular endothelial cells (HMEC-1).<br>In vitro chicken chorioallantoic membrane (CAM) assay.	DPSCs-CM significantly induced HMEC-1 migration and blood vessels formation in CAM assay.
**Ishizaka et al., 2013 [89]**	DPSCs-CM		In vitro culture of NIH3T3 cells and human neuroblastoma cell line TGW	DPSCs-CM triggered a potent anti-apoptotic activity of NIH3T3 cells and neurite outgrowth of human neuroblastoma cell line TGW.
**Mead et al., 2014 [90]**	DPSCs-CM	Retinal ganglion cells damage.	In vitro model of retinal ganglion cells damage in rats.	DPSCs-CM showed the presence of different neurotrophic factors, including NGF, BDNF and VEGF
**Paschalidis et al., 2014 [98]**	DPSCs-CM	Dental Pulp tissue	In vitro DPSCs culture.	DPSC's secretome raised DPSCs viability, migration and mineralization potential and totally offset TEGDMA-induced cytotoxicity.
**Hayashi et al., 2015 [104]**	DPSCs-CM	Pulp tissue	In vivo ectopic tooth transplantation model in a severe combined immunodeficiency mouse model.	DPSCs-CM transplantation yielded a larger volume of pulp regeneration, increased migration of endogenous cells from the surrounding tissue, elevated angiogenesis and decreased apoptosis in the regenerated pulp compared with the transplants of the CM from bone marrow and adipose cells, due to upregulation of CXCL14

			In vitro culture of mouse embryonic muscle myoblast cells (C2C12).	and MCP1 genes.<br>DPSCs-CM significantly increased cell migration, anti-apoptosis and angiogenesis in C2C12 cells.
**Murakami et al., 2015 [105]**	DPSCs-CM	Pulp tissue	In vitro model of pulp disease.	DPSCs-CM showed high angiogenic potential, induced neurite outgrowth and induced DPSCs odontoblastic differentiation.
**Ahmed et al, 2016 [92]**	DPSCs-CM	Alzheimer's disease (AD)	In vitro Alzheimer's disease model induced in human neuroblastoma SH-SY5Y cell line.	DPSCs secretome was rich in neurotrophic factors, antiapoptotic factors and Aβ-degrading enzyme (NEP). Thus, DPSCs are a potential source for the secretome-based treatment of Alzheimer's disease.
**Huang et al., 2016 [101]**	DPSCs- EXs	Pulp tissue	In vivo tooth root slice model implanted subcutaneously in the back of athymic nude mice.	DPSCs-EXs added to type I collagen membranes stimulated regeneration of dental pulp-like tissue.
			In vitro culture of DPSCs or HMSCs.	DPSCs-EXs activated the P38 MAPK pathway and upregulated the expression of genes required for odontogenic differentiation.
**Kawamura et al., 2016 [102]**	DPSCs-CM	Pulp tissue	In vivo ectopic tooth transplantation in severe combined immunodeficiency (SCID) mouse model.	The EDTA soluble chemical components and the DPSCs-CM reconstituted with the physical structure of autoclaved teeth, served as an inductive microenvironment, promoting cell proliferation, migration and odontoblastic differentiation.
**Yamamoto et al., 2016 [91]**	DPSCs-CM		In vitro culture of RT4-D6P2T cells.	DPSCs-CM significantly enhanced proliferation, migratory activity and significantly decreased the apoptosis of RT-D6P2T cells, as compared to supplementation of 5% FBS.

**Fujio et al., 2017 [99]**	DPSCs-CM	Bone healing	In vivo distraction osteogenesis (DO) healing mouse model.<br><br>In vitro HUVEC culture.	Local injection of hDPC-CM collected under hypoxic culture conditions enhanced angiogenesis and bone healing in the DO gap.<br><br>Hypoxic CM significantly had higher angiogenic potential than normoxic CM through the upregulation of angiogenic factors secreted from hDPCs.
**Gervois et al., 2017 [88]**	DPSCs-CM	Neurological disorders	In vitro culture of human SH-SY5Y neuroblastoma cells.	DPSCs-CM showed a significant chemoattractive effect on SH-SY5Y cells, stimulated neuronal maturation and neuritogenesis.
**Nakayama et al., 2017 [103]**	DPSCs-CM		In vitro culture of NIH3T3 cells, HUVECs and TGW cells	G-CSF mobilized DPSC-CM produced a significantly higher stimulatory effect on proliferation, migration and anti-apoptosis of NIH3T3 cells, immunomodulation, endothelial differentiation of HUVECs, and neurite extension in TGW cells, as compared to DPSCs-CM
**Song et al., 2017 [93]**	DPSCs-CM		In vitro culture of HUVECs	The number and total length of tubular structures per well in CM from hDPSCs increased significantly in HUVECs, as compared to hBMMSCs-CM
**Chen et al., 2019 [96]**	DPSCs-CM	Aneurysmal subarachnoid hemorrhage (aSAH)	In vivo rat aneurysmal subarachnoid hemorrhage (aSAH) model.	Early administration of DPSC-CM, particularly IGF-1, resulted in the improvement of microcirculation, tissue oxygenation, and neuroinflammation in the aSAH-injured brain.
**Makino et al., 2019 [95]**	DPSCs-CM	Nerves affected by Diabetic polyneuropathy	In vivo diabetic rat model.<br><br>In vitro HUVEC culture.	Single intramuscular injection of DPSC-CM into the hindlimb improved diabetic polyneuropathy, through enhancing sciatic nerve motor/sensory conduction velocity and blood flow as well as increasing intraepidermal nerve fiber density in the footpads of diabetic rats.<br><br>DPSC-CM significantly increased the in vitro proliferation of HUVECs.

**Wang et al., 2019 [94]**	DPSCs-CM	Amyotrophic lateral sclerosis (ALS)	In vivo mouse model of Amyotrophic Lateral Sclerosis (ALS).	Intraperitoneal injection of DPSC-CM significantly improved early neuromuscular junction (NMJ) innervation and increased motor neuron and NMJ preservation during late pre-symptomatic stages. Also, it significantly increased post-onset days of survival and overall lifespan.
**GMSCS-CM**				
**Shi et al., 2017 [112]**	GMSCs-EXs	Skin repair	In vivo diabetic rat skin defect model.	Incorporation of GMSC-derived exosomes to chitosan/silk hydrogel supported healing of skin defect in diabetic rats via re-epithelialization, collagen deposition and remodeling and enhanced angiogenesis and neuronal ingrowth.
**Rajan et al, 2017 [111]**	GMSCs-CM	Spinal motor neuron degenerative diseases.	In vitro mechanically injured murine motor-neuron-like NSC-34 cells culture.	GMSCs-CM offered neuroprotection via suppression of neural cells apoptosis, oxidative stress and inflammation achieved by suppressing TNF-α, SOD-1, iNOS, cleaved caspase-3, and Bax as well as by upregulating neurotrophic factors, such as BDNF, NGF, NT3, IL-10 and TGF-β.
**Diomede et al, 2018 [113]**	GMSCs-CM	Bone	In vivo rat calvarial defect model.<br><br>In vitro hGMSCs culture	3D-PLA scaffold enriched with hGMSCs and CM could improve osteogenesis in vitro as evident by mineralization and upregulation of osteogenesis-related genes. In addition to in vivo induction of new bone formation and osseointegration.
**Diomede et al, 2018 [116]**	GMSCs-EVs	Bone	In vivo rat calvarial defect model.<br><br>In vitro hGMSCs culture.	In vivo, 3D-PLA + PEI-EVs + hGMSCs and 3D-PLA + PEI-EVs scaffolds improved bone healing by showing better osteogenic properties. In vitro, 3D-PLA + PEI-EVs + hGMSCs exhibited greater osteogenic inductivity.
**Mao et al., 2019 [109]**	GMSCs-EVs	Peripheral nerve injuries	In vivo crush-injured sciatic nerve mouse model.<br><br>In vitro rat Schwann cell line RT4-D6P2T culture.	GMSC-EVs promoted axonal regeneration and functional recovery of injured mice sciatic nerves. GMSC- EVs promoted in vitro proliferation, migration and upregulation of dedifferentiation/repair genes (c-JUN, Notch1,

				GFAP and SOX2) of Schwann cells.
**Rao et al, 2019 [110]**	GMSCs-EXs	Peripheral (Sciatic) nerve injury	Injured sciatic nerve in the right hind limb in rats<br><br>In vitro co-culture with Schwann cells and Dorsal Root Ganglion (DRG) cells culture	Chitin conduit combined with GMSC-EXs significantly enhanced the number and diameter of nerve fibers and promoted myelin formation. Also, recovery of muscle function, nerve conduction function and motor function were observed.<br><br>GMSC-EXs significantly enhanced Schwann cell proliferation and DRG cells axon growth.
**Zhang et al., 2019 [114]**	GMSCs-EXs	Tongue	In vivo critical-sized tongue defect model in rats.	Small intestinal submucosa extracellular matrix with GMSCs or -GMSC-Exos constructs promoted tongue lingual papillae recovery and taste bud regeneration and reinnervation.
		**PDLSCS-CM**		
**Rajan et al., 2016[117]**	PDLSCs-CM PDLSCs-EVs	Multiple sclerosis (MS)	In vivo experimental autoimmune encephalomyelitis (EAE) mouse model of MS.	Intravenous infusion of PDLSCs-CM and PDLSCs-EMVs reduced clinical disease scores, augmented spine density and remyelination and attenuated apoptosis in the spinal cord of EAE mice. Also, it promoted anti-inflammatory and immunosuppressive effects, in the spinal cord and spleen of EAE mice. Such therapeutic effect was due to the reduction of pro-inflammatory cytokines IL-17, IL-1β, IL-6, IFN-γ, TNF-α and stimulation of anti-inflammatory IL-10. In addition to the downregulation of apoptosis-related genes STAT1, p53, Caspase 3 and Bax.
**Giacoppo et al., 2017 [119]**	PDLSCs-CM	Multiple Sclerosis (MS)	In vivo experimental autoimmune encephalomyelitis (EAE) mouse model of MS.	Intravenous injection of hPDLSC-CM under hypoxic conditions diminished clinical and histologic disease score caused by marked expression of anti-inflammatory cytokine IL-37

			In vitro injury model of NSC-34 neurons induced by mechanical scratching.	and the suppression of pro-inflammatory cytokines. hPDLSCs-CM showed the ability to modulate inflammatory, oxidative stress and apoptotic pathways.
**Rajan et al., 2017 [118]**	PDLSCs-CM PDLSCs-EVs	Multiple sclerosis (MS)	In vivo experimental autoimmune encephalomyelitis (EAE) mouse model of MS.	EAE mice administered with IV infusion of hPDLSCs-CM and EMVs showed significant improvement. The immunosuppressive role is suggested to be attributed to the presence of soluble immunomodulatory factors, NALP3 inflammasome inactivation and NF-κB reduction.
**Nagata et al., 2017 [122]**	PDLSC-CM	Periodontal disease	In vivo rat periodontal defect model	PDLSC-CM contained extracellular matrix proteins, enzymes, growth factors, angiogenic factors and cytokines as well as downregulated TNF-α expression. Thus enhanced periodontal regeneration.
**Diomede et al., 2018 [121]**	PDLSC-CM PDLSCs-EVs	Bone	In vitro PDLSCs culture<br><br>In vivo rats clavarial defect model	Collagen membrane enriched with PDLSCs and PEI-EVs showed high osteogenic potential, upregulation of osteogenesis-related genes in vitro and in vivo and enhanced osseous regeneration and osseointegration processes in vivo.
**Pizzicannella et al., 2019 [120]**	PDLSC-CM PDLSCs-EVs	Bone	In vitro PDLSCs culture.<br><br>In vivo rat calvarial defect model.	3D-COL enriched with PDLSCs and CM or EVs or PEI-EVs showed an increased expression of osteogenic markers, VEGF and VEGFR2. Enhanced osseous regeneration, vascularization and osseointegration of rat calvarial defects were revealed.
		**DFSCS-CM & SCAP-CM**		
**Kumar et al., 2017[123]**	DPSCs-CM, DFSCs-CM SCAP-CM	Neural differentiation	In vitro culture of IMR-32 pre-neuroblastic cell line	DMSCs secretomes significantly enhanced neural differentiation. DPSC secretome showed upregulation of the cytokines like GCSF, IFNγ,

			and TGFβ that promoted neural differentiation. DPSCs and their secretomes can be a source for cell-based and cell-free therapy for neural disorders and injury.
**Kumar et al., 2017 [100]**	DPSCs-CM<br>DFSCs-CM<br>SCAP-CM	In vitro culture of DMSCs (DPSCs, DFSCs and SCAP).	DMSC-CM demonstrated the presence of hepatic lineage proteins related to cell migration, cell survival and hepatic regeneration such as Adenomatous polyposis coli protein, growth arrest-specific protein 6 and Oncostatin M.
**Kumar et al, 2018 [124]**	DPSCs-CM<br>DFSCs-CM<br>SCAP-CM	In vitro culture of DMSCs (DPSCs, DFSCs and SCAP).	DMSC-CM, especially DPSC, increased osteogenic differentiation, as evident by enhanced mineralization, expression of ALP, DSPP staining and upregulation of osteogenic genes while decreased adipogenic differentiation.
